# Stochastic dynamics of Chikungunya virus infection model incorporating general incidence rate and immune responses

**DOI:** 10.1016/j.idm.2025.11.007

**Published:** 2025-11-19

**Authors:** Jingze Ma, Yan Wang

**Affiliations:** College of Science, China University of Petroleum (East China), Qingdao, Shandong, 266580, China

**Keywords:** CHIKV infection, Stochastic differential equations, Ornstein-Uhlenbeck process, Stationary distribution, Probability density function

## Abstract

This study investigates a stochastic model of Chikungunya virus (CHIKV) infection that incorporates a general incidence rate along with B-cell and CTL immune responses. Stochasticity is modeled through a log-normal Ornstein-Uhlenbeck process. We first establish the existence of a unique and globally positive solution. Then, the solution's dynamic behavior around the two steady states is examined, and it is shown that the stochastic model's dynamics at the steady state generalizes the global asymptotic stability of the deterministic model. We prove the existence of the stationary distribution by constructing suitable Lyapunov functions when the stochastic reproduction number is greater than one. The probability density function near the quasi-steady state is subsequently derived. Sufficient conditions for CHIKV extinction are provided by spectral radius analysis. Furthermore, we conduct uncertainty and sensitivity analyses to investigate the effects of key parameters on each population and the value of the stochastic reproduction number. Finally, numerical simulations are carried out to explore the impact of noise intensity and the average incidence rate on the dynamic behavior of the model.

## 1 Introduction

Chikungunya fever originated in Africa in 1952 and is caused by infection with the Chikungunya virus (CHIKV). The disease predominantly circulates in Africa, Asia, and the Indian subcontinent, but its transmission range has expanded in recent years owing to factors such as globalization and climate change. The symptoms of Chikungunya fever include high fever, arthritis, muscle pain, headache, occasional skin and gastrointestinal discomfort. These symptoms resemble those of dengue fever, leading to frequent misdiagnosis in endemic areas (Thiberville et al., 2013; da Cunha & Trinta, 2017; da Silva-Jnior et al., 2017). Despite its relatively low mortality rate (approximately 0.5–1.3 deaths per 1000 people), CHIKV still possesses significant epidemic potential (de Souza et al., 2024; Ng & Hapuarachchi, 2010). Currently, no specific drugs or effective vaccines are available against this virus (Bardach et al., 2025; Wilder-Smith & Wilder-Smith, 2024). Therefore, research on CHIKV infection is crucial.

CHIKV is primarily transmitted through Aedes mosquitoes, during which the virus is injected into the skin when an infected female mosquito feeds on the blood. Initially, the virus mainly replicates in dermal fibroblasts and macrophages, causing tissue damage and stimulating the release of pro-inflammatory cytokines (Bartholomeeusen et al., 2023; Gonsalves et al., 2025). Approximately one week later, the adaptive immune response begins to emerge, primarily consisting of B-cell-mediated humoral immunity and cytotoxic T lymphocyte (CTL)-mediated cellular immunity. The former generates antibodies against CHIKV antigens, while the latter eliminates virus-infected cells (Cunha et al., 2020; Lum et al., 2013; Lum & Ng, 2015).

In recent years, some scholars have begun to study mathematical models of CHIKV infection. Inspired by the host's intra-cellular immune response model for the dengue virus proposed by Nowak in literature (Nowak and May, 2000), Wang and Liu (Wang & Liu, 2017) constructed a four-dimensional mathematical model to describe the within-host CHIKV infection process. This model encompasses target cells, infected cells, CHIKV particles, and B-cell-mediated immune response, while also exploring the impact of immune delay on viral dynamics. Subsequently, Elaiw et al. extended this model by incorporating latent infection. Most recently, based on literature (Wang & Liu, 2017), Alade et al. (Alade et al., 2023) introduced CTL-mediated cellular immune response to establish a five-dimensional CHIKV infection model, aiming to investigate the effects of two types of immune responses on model dynamics. In light of these findings, we will also consider both B-cell and CTL immune responses simultaneously to better reflect actual infection scenarios.

Although deterministic models have been widely used to study the dynamic behavior of biological phenomena, their prediction results often deviate from reality due to the neglect of the randomness in biological processes. The process of viral attack on a host is influenced by various stochastic factors, such as fluctuations in the incidence rate between susceptible cells and virus particles, as well as the impact of environmental temperature and humidity on cell activity. Therefore, it is reasonable to adopt a stochastic model. Consequently, the introduction of a stochastic perturbation factor into the deterministic model is particularly important.

Stochastic models have become widely applied in the study of viral infection dynamics. Li et al. (Li et al., 2015) constructed a stochastic model for influenza A and confirmed the existence of the stochastic basic reproduction number, indicating that ignoring the impact of environmental noise would alter this threshold parameter. Mahrouf et al. (Mahrouf et al., 2017) studied the impact of environmental fluctuations on the dynamical behavior of viral infection models with general transmission rates. The research found that when environmental noise is large, the state variables experience significant fluctuations, making the use of stochastic models more accurate in describing the virus dynamics. Feng et al. (Feng et al., 2019) constructed a stochastic HIV-1 infection model with degenerate diffusion and studied the asymptotic dynamics of the stochastic model. The results highlighted the role of environmental noise in virus infection.

Currently, there are two main methods for introducing stochastic perturbation into a deterministic model: linear perturbation (Beddington & May 1977; Cai et al., 2017; Dalal et al., 2008; Du et al., 2020; Feng et al., 2019; Wang et al., 2017), and mean-reverting Ornstein-Uhlenbeck (OU) process (Allen, 2016; Cai et al., 2018; Cai et al., 2018, 2018; Mamis & Farazmand, 2023; Wu et al., 2024; Zhou et al., 2022; Zhou et al., 2022, 2022). Compared with linear white noise models, the OU process, with its advantages of continuity, non-negativity, practicality, and asymptotic distribution characteristics, is more suitable for describing the stochastic fluctuations of key parameters in biological and experimental environments (Allen, 2016). Recent research has shown that in models of the COVID-19 virus, the OU process provides a close fit to real-world data than linear perturbation models (Mamis & Farazmand, 2023). Therefore, we incorporate the OU process into the deterministic model to reflect the perturbation factor.

In this study, we establish a stochastic CHIKV model, incorporating a general incidence rate, B-cell-mediated humoral immunity, CTL-mediated cellular immunity, and a log-mean-reverting OU process. To the best of our knowledge, few studies on stochastic CHIKV models have integrated both types of immune response and general incidence rate. This pose challenges for the theoretical analysis, such as the existence of the stationary distribution. Compared to the existing models, our main contributions are as follows:

• We integrate the general incidence rate and OU process into the existing deterministic CHIKV model to establish the stochastic model, providing deeper insights into the infection dynamics of the virus.
• We rigorously derive stochastic behavior at steady state and identify the indicator linking the asymptotic behavior of the stochastic model to the global asymptotic stability of the deterministic model. Additionally, an approximate expression for the probability density function at the quasi-steady state is derived.
• A novel approach for constructing appropriate Lyapunov functions is proposed, proving that the stationary distribution exists if the stochastic reproduction number of the system is greater than one.
• By utilizing the spectral radius analysis method, a critical condition for extinction is obtained, thereby proving that the infected cells and virus tend to extinction at an exponential rate.
• Using Latin Hypercube Sampling and Partial Rank Correlation Coefficient methods, we perform uncertainty and sensitivity analysis to investigate the effects of key parameters on each population, as well as the stochastic reproduction number.
• We investigate the effects of noise intensity, the reversion rate, and the average incidence rate on the dynamic behavior of the model and find that increasing the reversion rate, decreasing the noise intensity, and reducing the average incidence rate can accelerate CHIKV extinction.

The remainder of this paper is organized as follows. In Section 2, we establish a deterministic CHIKV model with two immune responses and general incidence rate, and then introduce the logarithmic OU process to construct the stochastic model. We further derive the existence and uniqueness of the global solution. A theoretical analysis of the dynamic behavior at the steady state of the stochastic model is presented in Section 3. In Section 4, we establish the stochastic reproduction number for the existence of the stationary distribution by constructing appropriate Lyapunov functions, and also present sufficient conditions for virus elimination. Section 5 presents an approximate expression of the probability density function around the quasi-steady state. In Section 6, we employ uncertainty and sensitivity analysis to identify the key parameters that influence the population size and the value of the stochastic reproduction number. The numerical simulations in Section 7 verify our theoretical findings and explore the influence of noise intensity and the average incidence rate on the dynamics of the model. Finally, we conclude our results and outline directions for future research.

## 2 Model formulation

### 2.1 The deterministic model

Recently, a CHIKV infection model involving B-cell and CTL immune responses is presented in the following ordinary differential equations (Alade et al., 2023),

(1) $$\left\{\begin{array}{l} \frac{dS}{dt}=\lambda -dS-bSV,\;\\ \frac{dI}{dt}=bSV-aI-\epsilon IZ,\;\\ \frac{dV}{dt}=mI-rV-qVB,\;\\ \frac{dB}{dt}=\eta +cBV-\delta B,\;\\ \frac{dZ}{dt}=\gamma +\omega IZ-\mu Z,\; \end{array}\right)$$

where *S*, *I*, *V*, *B* and *Z* represent the concentrations of target cells, infected cells, CHIKV particles, B-cells, and CTLs, respectively. The target cells include dermal fibroblasts and macrophages (Bartholomeeusen et al., 2023). The meanings of the other parameters are listed in Table 1.

**Table 1:** Parameter meanings of system (1).

Parameter	Interpretation
*λ*	Constant input rate
*d*	Death rate of target cells
*b*	Incidence rate between target cells and CHIKV
*a*	Death rate of infected cells
*m*	Multiplication rate of CHIKV particles
*r*	Clearance rate of free CHIKV particles
*q*	B-cell effectiveness
*η*	Production rate of B-cells
*c*	B-cell responsiveness
*δ*	Death rate of B-cells
*ϵ*	CTL effectiveness
*γ*	Production rate of CTLs
*ω*	CTL responsiveness
*μ*	Death rate of CTLs

The incidence rate *bSV* described in model (1) is bilinear. In this case, the incidence rate is proportional to the product of the virus and the concentration of the target cells (Elaiw et al., 2024). However, as the virus particles spread within the host, certain interventions are typically implemented to prevent the virus from growing infinitely. In viral dynamics models, the use of a saturated incidence rate, $\frac{bSV}{1+m_{1}V}$, can describe the inhibitory effect on target cells when the number of virus-infected cells becomes sufficiently large, where *m*_1_ reflects the level of inhibition (Li & Ma, 2007). Studies (Alade, 2021a; Alade et al., 2021a, 2021b) have shown that incorporating general incidence rate into the CHIKV infection model can include not only bilinear and saturated incidence but also a wider variety of other forms. This approach not only enhances the flexibility and adaptability of the model but also makes it more general and universal, enabling it to better explain and predict the transmission dynamics of CHIKV under different scenarios.

**Fig. 1:**
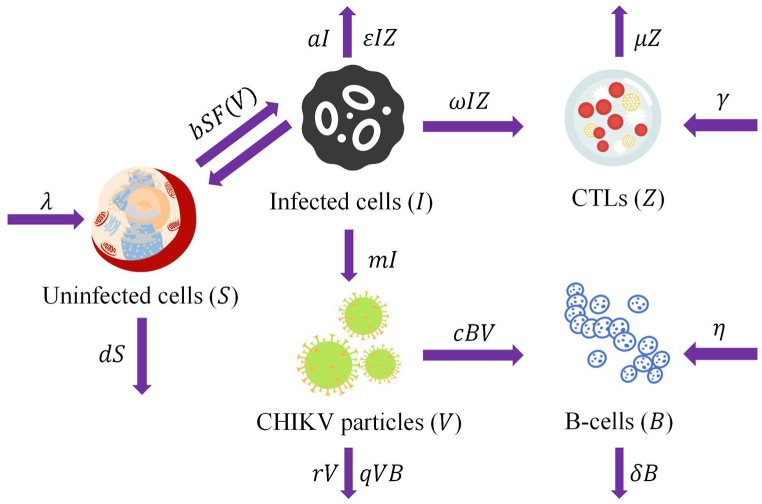
CHIKV infection flowchart.

Based on model (1) and the flowchart for CHIKV infection process (see Fig. 1), we establish a deterministic model featuring the general incidence rate and incorporating two types of immune responses as follows:

(2) $$\left\{\begin{array}{l} \frac{dS}{dt}=\lambda -dS-bSF\left(V\right),\;\\ \frac{dI}{dt}=bSF\left(V\right)-aI-\epsilon IZ,\;\\ \frac{dV}{dt}=mI-rV-qVB,\;\\ \frac{dB}{dt}=\eta +cBV-\delta B,\;\\ \frac{dZ}{dt}=\gamma +\omega IZ-\mu Z,\; \end{array}\right)$$

where *F*(*V*) is a continuous and first-order differentiable function of *V* that satisfies the following conditions:

$\left(A_{1}\right)F\left(0\right)=0$;

$\left(A_{2}\right)\;F^{\prime }\left(V\right)\geq 0$ for *V* ≥ 0;

$\left(A_{3}\right)0\leq {\left(\frac{V}{F\left(V\right)}\right)}^{\prime }\leq K_{0}$, where *K*_0_ is a positive constant.

For system (2), according to the next generation method (van den Driessche & Watmough, 2002), we obtain the basic reproduction number as

$$R_{0}=\frac{bm\delta \lambda \mu F^{\prime }\left(0\right)}{d\left(r\delta +q\eta \right)\left(a\mu +\epsilon \gamma \right)}.$$

Through calculation, under conditions $\left(A_{1}\right)$ and $\left(A_{2}\right)$, we obtain the existence of the steady states of system (2) as follows:

• An infection-free steady state $Q_{0}\left(S_{0},0,0,B_{0},Z_{0}\right)$ always exists, where $S_{0}=\frac{\lambda }{d},\;B_{0}=\frac{\eta }{\delta },\;Z_{0}=\frac{\gamma }{\mu }$.
• If *R*_0_ > 1, there exists a unique infection steady state $Q^{*}\left(S^{*},I^{*},V^{*},B^{*},Z^{*}\right)$, where

(3) $$\begin{array}{l} S^{*}=\frac{r\left(\delta -cV^{*}\right)+q\eta }{bmF\left(V\right)\left(\delta -cV^{*}\right)}\cdot \frac{a\omega V^{*}\left[rcV^{*}-\left(r\delta +q\eta \right)\right]+m\left(a\mu +\gamma \epsilon \right)\left(\delta -cV^{*}\right)}{\omega V^{*}\left[rcV^{*}-\left(r\delta +q\eta \right)\right]+m\mu \left(\delta -cV^{*}\right)},\\ I^{*}=\frac{\left[r\left(\delta -cV^{*}\right)+q\eta \right]V^{*}}{m\left(\delta -cV^{*}\right)},\;B^{*}=\frac{\eta }{\delta -cV^{*}},\\ Z^{*}=\frac{m\gamma \left(\delta -cV^{*}\right)}{\omega V^{*}\left[rcV^{*}-\left(r\delta +q\eta \right)\right]+m\mu \left(\delta -cV^{*}\right)}. \end{array}$$

Substituting the equalities (3) into *λ* − *dS*∗ − *bS*∗*F*(*V*∗) = 0, we obtain an equation for *V*∗ and then solve for the positive solution *V*∗. We know that with a certain invariant set, if the positive steady state is globally asymptotically stable, there can not exist any other positive steady state in the invariant set. The reason is that global asymptotic stability requires that all system trajectories starting from any initial state, eventually converge to this steady state. If there were another distinct steady state, the trajectories starting near that point would not be able to converge to the original steady state, which would contradict the definition of global asymptotic stability. In Corollary 3.2 of Section 3, we have rigorously proven the global asymptotic stability of *Q*∗. Therefore, *Q*∗ must be the unique infection steady state of system (2).

### 2.2 The stochastic model

The CHIKV incidence rate in host cells can be influenced by various random disturbances. Studies have shown that host cell surface adhesion molecules, such as the MXRA8 receptor, directly bind to chikungunya virus particles, which enhances virus attachment and internalization within cells (Mandary et al., 2019; Zhang et al., 2018). The expression level of MXRA8 receptor varies significantly between cells, and this heterogeneity directly affects the efficiency of viral binding (Gonsalves et al., 2025; Zhang et al., 2018). Simultaneously, the host genetic diversity of CHIKV variants leads to differences in the virulence of the virus. Low-fidelity variants result in reduced replication rates and pathogenicity (Mandary et al., 2019). Meanwhile, environmental temperature can affect the efficacy of key antiviral mediators, such as IFN-*α*/*β* (Lane et al., 2018). In addition, newly produced viral particles contain a portion of defective viral particles. Although these defective particles lack independent replication ability, they can competitively bind to target cell receptors, thereby dynamically reducing the probability of successful contact by infectious viral particles (Vignuzzi & Lpez, 2019). Therefore, the interaction between CHIKV and host cells is random and complex.

Motivated by published literature (Allen, 2016; Dong et al., 2025; Liu & Jiang, 2024; Shi & Jiang, 2023), we adopt a logarithmic mean-reverting OU process to simulate the random disturbance of the incidence rate *b* and introduce the following stochastic differential equation:

$$d\left(\ln\;b\right)=\rho \left(\ln\;\bar{b}-\ln\;b\right)\;dt+\sigma \;dB\left(t\right),$$

where the term $\ln\;\bar{b}$ represents the logarithmic form of the long-term average incidence rate, *ρ* > 0 is the reversion rate, *σ* > 0 is the noise intensity, and B(*t*) is a standard Brownian motion defined on a complete probability space. Let $x\left(t\right)=\ln\;b\left(t\right)-\ln\;\bar{b}$ and $b=\bar{b}e^{x}$, then *x*(*t*) satisfies the OU stochastic differential equation:

(4) dx(t)=−ρxdt+σdB(t).

Then, we obtain the following stochastic differential equations:

(5) $$\left\{\begin{array}{l} dS=\left[\lambda -dS-\bar{b}e^{x}SF\left(V\right)\right]dt,\;\\ dI=\left[\bar{b}e^{x}SF\left(V\right)-aI-\epsilon IZ\right]dt,\;\\ dV=\left(mI-rV-qVB\right)dt,\;\\ dB=\left(\eta +cBV-\delta B\right)dt,\;\\ dZ=\left(\gamma +\omega IZ-\mu Z\right)dt,\;\\ dx=-\rho x\;dt+\sigma \;dB\left(t\right).\; \end{array}\right)$$

For simplicity, we define $X\left(t\right)=\left(S\left(t\right),I\left(t\right),V\left(t\right),B\left(t\right),Z\left(t\right),x\left(t\right)\right)$, *X*_0_ = (*S*(0), *I*(0), *V*(0), *B*(0), *Z*(0), *x*(0)) and $X=\left(X_{1},X_{2},X_{3},X_{4},X_{5},X_{6}\right)\in R_{+}^{5}\times R$, where $R_{+}$ denotes the set of positive real numbers. L denotes the differential operator associated with It$\hat{o}$’s formula. Moreover, we denote the indicator function of set A as $1_{A}$. When **I** represents a vector or matrix, **I**^T^ denotes its transpose and **I**^−1^ indicates its inverse.

Theorem 2.1 *For any initial value* *X*_0_*, under conditions* $\left(A_{1}\right)-\left(A_{3}\right)$ *system* (5) *has a unique solution* $X\left(t\right)\in R_{+}^{5}\times R$ *for* *t* ≥ 0 *almost surely (a.s.).*

The proof of Theorem 2.1 is given in Appendix A.

According to system (5), we have

$$\begin{array}{l} \frac{dS}{dt}=\lambda -dS-\bar{b}e^{x}SV\leq \lambda -dS,\\ \frac{d\left(S+I\right)}{dt}=\lambda -dS-aI-\epsilon IZ\leq \lambda -dS-aI,\\ \frac{dV}{dt}=mI-rV-qVB\leq mI-rV,\\ \frac{dB}{dt}=\eta +cBV-\delta B\geq \eta -\delta B,\\ \frac{dZ}{dt}=\gamma +\omega IZ-\mu Z\geq \gamma -\mu Z. \end{array}$$

Thus, we obtain

$$\begin{array}{l} S\left(t\right)\leq \frac{\lambda }{d}+e^{-dt}\left[S\left(0\right)-\frac{\lambda }{d}\right],\;S\left(t\right)+I\left(t\right)\leq M_{1}+e^{-\left(d\wedge a\right)t}\left[S\left(0\right)+I\left(0\right)-M_{1}\right],\\ V\left(t\right)\leq M_{2}+e^{-rt}\left[V\left(0\right)-M_{2}\right],\;B\left(t\right)\geq \frac{\eta }{\delta }+e^{-\delta t}\left[B\left(0\right)-\frac{\eta }{\delta }\right],\;Z\left(t\right)\geq \frac{\gamma }{\mu }+e^{-\mu t}\left[Z\left(0\right)-\frac{\gamma }{\mu }\right]. \end{array}$$

If $S\left(0\right)\leq \frac{\lambda }{d}$, then $S\left(t\right)\leq \frac{\lambda }{d}$ for sufficiently large time *t*. Similarly, if *S*(0) + *I*(0) ≤ *M*_1_, *V*(0) ≤ *M*_2_, $B\left(0\right)\geq \frac{\eta }{\delta }$, and $Z\left(0\right)\geq \frac{\gamma }{\mu }$, then *S*(*t*) + *I*(*t*) ≤ *M*_1_, *V*(*t*) ≤ *M*_2_, $B\left(t\right)\geq \frac{\eta }{\delta }$, and $Z\left(t\right)\geq \frac{\gamma }{\mu }$ for sufficiently large time *t*, where

(6) $$M_{1}=\frac{\lambda }{d\wedge a},\;M_{2}=\frac{m}{r}M_{1}=\frac{m\lambda }{r\left(d\wedge a\right)}.$$

Applying It$\hat{o}$’s formula (Mao, 2008), we have

$$\begin{array}{ll} L\left(S+I+\frac{a}{2m}V+\frac{aq}{2mc}B+\frac{\epsilon }{\omega }Z\right) & =\lambda +\frac{aq\eta }{2mc}+\frac{\epsilon \gamma }{\omega }-dS-\frac{a}{2}I-\frac{ar}{2m}V-\frac{aq\delta }{2mc}B-\frac{\epsilon \mu }{\omega }Z\\ & \leq \lambda +\frac{aq\eta }{2mc}+\frac{\epsilon \gamma }{\omega }-\alpha \left(S+I+\frac{a}{2m}V+\frac{aq}{2mc}B+\frac{\epsilon }{\omega }Z\right), \end{array}$$

where $\alpha =\min\left\{d,\frac{a}{2},r,\delta ,\mu \right\}$. If $S\left(0\right)+I\left(0\right)+\frac{a}{2m}V\left(0\right)+\frac{aq}{2mc}B\left(0\right)+\frac{\epsilon }{\omega }Z\left(0\right)\leq \frac{\lambda }{\alpha }+\frac{aq\eta }{2mc\alpha }+\frac{\epsilon \gamma }{\omega \alpha }$, then

$$S\left(t\right)+I\left(t\right)+\frac{a}{2m}V\left(t\right)+\frac{aq}{2mc}B\left(t\right)+\frac{\epsilon }{\omega }Z\left(t\right)\leq \frac{\lambda }{\alpha }+\frac{aq\eta }{2mc\alpha }+\frac{\epsilon \gamma }{\omega \alpha }$$

for sufficiently large time *t*, which indicates

(7) $$B\left(t\right)\leq \frac{2mc}{aq}\left(\frac{\lambda }{\alpha }+\frac{aq\eta }{2mc\alpha }+\frac{\epsilon \gamma }{\omega \alpha }\right)≔M_{3},\;Z\left(t\right)\leq \frac{\omega }{\epsilon }\left(\frac{\lambda }{\alpha }+\frac{aq\eta }{2mc\alpha }+\frac{\epsilon \gamma }{\omega \alpha }\right)≔M_{4}$$

for sufficiently large time *t*. Therefore, system (5) has a positive invariant set

$$\Gamma =\left\{X\in R_{+}^{5}\times R|S\leq \frac{\lambda }{d},\;S+I\leq M_{1},\;V\leq M_{2},\;\frac{\eta }{\delta }\leq B\leq M_{3},\;\frac{\gamma }{\mu }\leq Z\leq M_{4}\right\}.$$

In the subsequent analysis, we assume that the initial value of system lies within the invariant set, and we study the dynamic behavior of system (5) within this set.

## 3 Dynamic properties at the steady state

In this section, we explore the dynamic property of the solution to the stochastic model (5) near the infection-free steady state *Q*_0_ and infection steady state *Q*∗ of its corresponding deterministic model (2).

### 3.1 Dynamic property around the infection-free steady state

Theorem 3.1 *Under conditions* $\left(A_{1}\right)$ *and* $\left(A_{3}\right)$*, if* *R*_0_ < 1*, for any given initial condition* *X*_0_ ∈ Γ*, the solution of model* (5) *has the following property:*

$${\mathrm{lim sup}}_{t\to \infty }E\left[\frac{1}{t}\int_{0}^{t}\left({\left(S\left(\tau \right)-S_{0}\right)}^{2}+{\left(B\left(\tau \right)-B_{0}\right)}^{2}+{\left(Z\left(\tau \right)-Z_{0}\right)}^{2}+I^{2}\left(\tau \right)+V^{2}\left(\tau \right)\right)d\tau \right]\leq \frac{\lambda F^{\prime }\left(0\right)M_{2}\bar{b}}{dm_{1}}{\left(e^{\frac{\sigma^{2}}{\rho }}-2e^{\frac{\sigma^{2}}{4\rho }}+1\right)}^{\frac{1}{2}},$$

*where the expression for* *m*_1_ *is given by* (11).

*Proof*. We introduce a function *H*(*X*) = *x* − ln *x* − 1. Clearly, *H*(*x*) ≥ 0 for *x* > 0, and *H*(*x*) = 0 if and only if *x* = 1. Define

$$P_{1}=S_{0}H\left(\frac{S}{S_{0}}\right)+I+\frac{a\mu +\epsilon \gamma }{m\mu }V+\frac{q\left(a\mu +\epsilon \gamma \right)}{m\mu c}B_{0}H\left(\frac{B}{B_{0}}\right)+\frac{\epsilon }{\omega }Z_{0}H\left(\frac{Z}{Z_{0}}\right).$$

Applying It$\hat{o}$’s formula (Mao, 2008) and inequality (A.2), we get

$$\begin{array}{l} LP_{1}=\left(1-\frac{S_{0}}{S}\right)\left[\lambda -dS-\bar{b}e^{x}SF\left(V\right)\right]+\bar{b}e^{x}SF\left(V\right)-aI-\epsilon IZ+\frac{a\mu +\epsilon \gamma }{m\mu }\left(mI-rV-qVB\right)\\ \;\;\;\;\;\;\;\;\;\;\;\;+\frac{q\left(a\mu +\epsilon \gamma \right)}{m\mu c}\left(1-\frac{B_{0}}{B}\right)\left(\eta +cBV-\delta B\right)+\frac{\epsilon }{\omega }\left(1-\frac{Z_{0}}{Z}\right)\left(\gamma +\omega IZ-\mu Z\right)\\ \;\;\;\;\;\;\;\;\leq \left(1-\frac{S_{0}}{S}\right)\left(dS_{0}-dS\right)+bS_{0}F\left(V\right)-\frac{\left(a\mu +\epsilon \gamma \right)rV}{m\mu }-\frac{\left(a\mu +\epsilon \gamma \right)qB_{0}V}{m\mu }\\ \;\;\;\;\;\;\;\;\;\;\;\;+\frac{q\left(a\mu +\epsilon \gamma \right)}{m\mu c}\left(1-\frac{B_{0}}{B}\right)\left(\delta B_{0}-\delta B\right)+\frac{\epsilon }{\omega }\left(1-\frac{Z_{0}}{Z}\right)\left(\mu Z_{0}-\mu Z\right)+\bar{b}e^{x}S_{0}F^{'}\left(0\right)V-\bar{b}S_{0}F^{'}\left(0\right)V\\ \;\;\;\;\;\;\;\;\leq -d\frac{{\left(S-S_{0}\right)}^{2}}{S}-\frac{q\delta \left(a\mu +\epsilon \gamma \right)}{m\mu c}\frac{{\left(B-B_{0}\right)}^{2}}{B}-\frac{\epsilon \mu }{\omega }\frac{{\left(Z-Z_{0}\right)}^{2}}{Z}+\frac{\left(r\delta +q\eta \right)\left(a\mu +\epsilon \gamma \right)}{\delta m\mu }\left(R_{0}-1\right)V\\ \;\;\;\;\;\;\;\;\;\;\;\;+\left|e^{x}-1\right|\bar{b}S_{0}F^{'}\left(0\right)V. \end{array}$$

Combine *R*_0_ < 1, one obtains that

(8) $$LP_{1}\leq -d\frac{{\left(S-S_{0}\right)}^{2}}{S_{0}}-\frac{q\left(a\mu +\epsilon \gamma \right)\delta }{m\mu c}\frac{{\left(B-B_{0}\right)}^{2}}{M_{3}}-\frac{\epsilon \mu }{\omega }\frac{{\left(Z-Z_{0}\right)}^{2}}{M_{4}}+\left|e^{x}-1\right|\bar{b}S_{0}F^{\prime }\left(0\right)M_{2}.$$

In view of system (5), one gets

$$L\left(S+I+\frac{\epsilon }{\omega }Z\right)=\lambda -dS-aI+\frac{\epsilon \gamma }{\omega }-\frac{\epsilon \mu }{\omega }Z=-d\left(S-S_{0}\right)-aI-\frac{\epsilon \mu }{\omega }\left(Z-Z_{0}\right).$$

Define

$$P_{2}=\frac{1}{2}{\left[S-S_{0}+I+\frac{\epsilon }{\omega }\left(Z-Z_{0}\right)\right]}^{2}.$$

By Young's inequality,

$$\pm AB\leq \frac{\beta }{2}A^{2}+\frac{1}{2\beta }B^{2},$$

where *A*, *B* ≥ 0, *β* > 0. For example,

$$-\left(a+d\right)I\left(S-S_{0}\right)\leq \frac{a}{4}I^{2}+\frac{{\left(a+d\right)}^{2}}{a}{\left(S-S_{0}\right)}^{2},$$

note that *β* is taken to be *a*/2. Thereafter,

$$\begin{array}{ll} LP_{2} & =-d{\left(S-S_{0}\right)}^{2}-aI^{2}-\frac{\epsilon^{2}\mu }{\omega^{2}}{\left(Z-Z_{0}\right)}^{2}-\left(a+d\right)I\left(S-S_{0}\right)-\frac{\epsilon \left(\mu +a\right)}{\omega }I\left(Z-Z_{0}\right)-\frac{\epsilon \left(\mu +d\right)}{\omega }\left(S-S_{0}\right)\left(Z-Z_{0}\right)\\ & \leq -d{\left(S-S_{0}\right)}^{2}-aI^{2}-\frac{\epsilon^{2}\mu }{\omega^{2}}{\left(Z-Z_{0}\right)}^{2}+\frac{1}{2}\cdot \frac{a}{2}I^{2}+\frac{{\left(a+d\right)}^{2}}{a}{\left(S-S_{0}\right)}^{2}+\frac{1}{2}\cdot \frac{a}{2}I^{2}\\ & \;\;\;\;+\frac{\epsilon^{2}{\left(\mu +a\right)}^{2}}{a\omega^{2}}{\left(Z-Z_{0}\right)}^{2}+\frac{d}{2}{\left(S-S_{0}\right)}^{2}+\frac{\epsilon^{2}{\left(\mu +d\right)}^{2}}{2d\omega^{2}}{\left(Z-Z_{0}\right)}^{2}\\ & =-\frac{a}{2}I^{2}+E_{1}{\left(S-S_{0}\right)}^{2}+E_{2}{\left(Z-Z_{0}\right)}^{2}, \end{array}$$

where

(9) $$E_{1}=\frac{2a^{2}+2d^{2}+3ad}{2a},\;E_{2}=\frac{\epsilon^{2}}{\omega^{2}}\left[\frac{{\left(\mu +d\right)}^{2}}{2d}+\frac{\mu^{2}+a^{2}+a\mu }{a}\right].$$

Let $\begin{array}{l} J_{1}=\min\left\{\frac{d}{2S_{0}E_{1}},\;\frac{\epsilon \mu }{2\omega M_{4}E_{2}}\right\}, \end{array}$ combine (8) and the above, we have

$$L\left(P_{1}+J_{1}P_{2}\right)\leq -\frac{d}{2S_{0}}{\left(S-S_{0}\right)}^{2}-\frac{q\delta \left(a\mu +\epsilon \gamma \right)}{m\mu cM_{3}}{\left(B-B_{0}\right)}^{2}-\frac{\epsilon \mu }{2\omega M_{4}}{\left(Z-Z_{0}\right)}^{2}-\frac{aJ_{1}}{2}I^{2}+\left|e^{x}-1\right|\bar{b}S_{0}F^{\prime }\left(0\right)M_{2}.$$

In addition, we get

$$L\frac{V^{2}}{2}=mIV-rV^{2}-qV^{2}B\leq -rV^{2}+\frac{r}{2}V^{2}+\frac{m^{2}}{2r}I^{2}=-\frac{r}{2}V^{2}+\frac{m^{2}}{2r}I^{2}.$$

Let $P=P_{1}+J_{1}P_{2}+\frac{arJ_{1}}{2m^{2}}\frac{V^{2}}{2}$, and then

(10) $$\begin{array}{l} LP\leq -\frac{d}{2S_{0}}{\left(S-S_{0}\right)}^{2}-\frac{q\delta \left(a\mu +\epsilon \gamma \right)}{m\mu cM_{3}}{\left(B-B_{0}\right)}^{2}-\frac{\epsilon \mu }{2\omega M_{4}}{\left(Z-Z_{0}\right)}^{2}-\frac{aJ_{1}}{4}I^{2}-\frac{ar^{2}J_{1}}{4m^{2}}V^{2}+\left|e^{x}-1\right|\bar{b}S_{0}F^{'}\left(0\right)M_{2}\\ \;\;\;\;\;\;\leq -m_{1}\left[{\left(S-S_{0}\right)}^{2}+{\left(B-B_{0}\right)}^{2}+{\left(Z-Z_{0}\right)}^{2}+I^{2}+V^{2}\right]+\left|e^{x}-1\right|\bar{b}S_{0}F^{'}\left(0\right)M_{2}, \end{array}$$

where

(11) $$m_{1}=\min\left\{\frac{d}{2S_{0}},\;\frac{q\delta \left(a\mu +\epsilon \gamma \right)}{m\mu cM_{3}},\;\frac{\epsilon \mu }{2\omega M_{4}},\;\frac{aJ_{1}}{4},\frac{ar^{2}J_{1}}{4m^{2}}\right\}.$$

Integrating both sides from 0 to *t* and taking the expectation, then dividing both sides by *t*, we have

(12) $$0\leq \frac{EP\left(X\left(t\right)\right)}{t}-\frac{EP\left(X_{0}\right)}{t}\leq -m_{1}E\left[\frac{1}{t}\int_{0}^{t}\left({\left(S\left(\tau \right)-S_{0}\right)}^{2}+{\left(B\left(\tau \right)-B_{0}\right)}^{2}+{\left(Z\left(\tau \right)-Z_{0}\right)}^{2}+I^{2}\left(\tau \right)+V^{2}\left(\tau \right)\right)d\tau \right]+\frac{\lambda F^{'}\left(0\right)M_{2}\bar{b}}{d}E\left[\frac{1}{t}\int_{0}^{t}|e^{x\left(\tau \right)}-1|d\tau \right].$$

Combining with inequality (B.2), and taking the superior limit on both sides of (12), we get

$${\mathrm{lim sup}}_{t\to \infty }E\left[\frac{1}{t}\int_{0}^{t}\left({\left(S\left(\tau \right)-S_{0}\right)}^{2}+{\left(B\left(\tau \right)-B_{0}\right)}^{2}+{\left(Z\left(\tau \right)-Z_{0}\right)}^{2}+I^{2}\left(\tau \right)+V^{2}\left(\tau \right)\right)d\tau \right]\leq \frac{\lambda F^{\prime }\left(0\right)M_{2}\bar{b}}{dm_{1}}{\left(e^{\frac{\sigma^{2}}{\rho }}-2e^{\frac{\sigma^{2}}{4\rho }}+1\right)}^{\frac{1}{2}}.$$

This completes this proof.

According to Theorem 3.1, if *R*_0_ < 1, we conclude that the solution of system (5) fluctuates around the infection-free steady state *Q*_0_. In particular, when the noise intensity is zero (*σ* = 0), inequality (10) can be simplified as

$$LP\leq -m_{1}\left[{\left(S-S_{0}\right)}^{2}+{\left(B-B_{0}\right)}^{2}+{\left(Z-Z_{0}\right)}^{2}+I^{2}+V^{2}\right].$$

The right hand term above is in the form of a negative-definite quadratic form. By the Lyapunov method, we obtain the following conclusion.

Corollary 3.1 *Under conditions* $\left(A_{1}\right)$ *and* $\left(A_{3}\right)$*, if* *R*_0_ < 1*, the infection-free steady state* $Q_{0}\left(S_{0},0,0,B_{0},Z_{0}\right)$ *of system* (2) *is globally asymptotically stable on the invariant set* Γ.

### 3.2 Dynamic property around the infection steady state

Theorem 3.2 *Under conditions* $\left(A_{2}\right)$ *and* $\left(A_{3}\right)$*, if* *R*_0_ > 1*, for any given initial condition* *X*_0_ ∈ Γ*, the solution* *X*(*t*) *of system* (5) *satisfies*

$$\begin{array}{ll} & {\mathrm{lim sup}}_{t\to \infty }E\left[\frac{1}{t}\int_{0}^{t}\left({\left(S\left(\tau \right)-S^{*}\right)}^{2}+{\left(I\left(\tau \right)-I^{*}\right)}^{2}+{\left(V\left(\tau \right)-V^{*}\right)}^{2}+{\left(B\left(\tau \right)-B^{*}\right)}^{2}+{\left(Z\left(\tau \right)-Z^{*}\right)}^{2}\right)d\tau \right]\\ & \;\;\leq \frac{S^{*}F^{\prime }\left(0\right)M_{2}\bar{b}}{m_{2}}{\left(e^{\frac{\sigma^{2}}{\rho }}-2e^{\frac{\sigma^{2}}{4\rho }}+1\right)}^{\frac{1}{2}}, \end{array}$$

*where the expression for* *m*_2_ *is given by* (15).

*Proof*. The infection steady state *Q*∗ of system (2) satisfies

(13) $$\begin{array}{l} \left\{\begin{array}{l} \lambda =dS^{*}+\bar{b}S^{*}F\left(V^{*}\right),\;\\ aI^{*}=\bar{b}S^{*}F\left(V^{*}\right)-\epsilon I^{*}Z^{*},\;\\ rV^{*}=mI^{*}-qV^{*}B^{*},\;\\ \delta B^{*}=\eta +cB^{*}V^{*},\;\\ \mu Z^{*}=\gamma +\omega I^{*}Z^{*}.\; \end{array}\right) \end{array}$$

Firstly, applying It$\hat{o}$’s formula, we calculate that

$$\begin{array}{ll} L\left(-S^{*}\ln\frac{S}{S^{*}}\right) & =S^{*}\left[-\frac{dS^{*}+\bar{b}S^{*}F\left(V^{*}\right)}{S}+d+\bar{b}e^{x}F\left(V\right)\right]\\ & =-\frac{S^{*}}{S}\left[-d\left(S-S^{*}\right)+\bar{b}\left(S^{*}F\left(V^{*}\right)-SF\left(V\right)\right)+\left(\bar{b}-\bar{b}e^{x}\right)SF\left(V\right)\right]\\ & \leq \frac{S^{*}}{S}d\left(S-S^{*}\right)+\bar{b}S^{*}F\left(V^{*}\right)\left[\frac{F\left(V\right)}{F\left(V^{*}\right)}-\frac{S^{*}}{S}\right]+\left|e^{x}-1\right|\bar{b}S^{*}F\left(V\right),\\ L\left(-I^{*}\ln\frac{I}{I^{*}}\right) & =-\bar{b}e^{x}SF\left(V\right)\frac{I^{*}}{I}+aI^{*}+\epsilon I^{*}Z\\ & =\bar{b}S^{*}F\left(V^{*}\right)\left[1-\frac{e^{x}SF\left(V\right)I^{*}}{S^{*}F\left(V^{*}\right)I}\right]+\epsilon I^{*}Z^{*}\left[\frac{Z}{Z^{*}}-1\right],\\ L\left(-V^{*}\ln\frac{V}{V^{*}}\right) & =mI^{*}\left(1-\frac{IV^{*}}{I^{*}V}\right)+qV^{*}B^{*}\left(\frac{B}{B^{*}}-1\right)\\ & \leq -mI^{*}\left(\ln\frac{I}{I^{*}}-\ln\frac{V}{V^{*}}\right)+qV^{*}B^{*}\left(\frac{B}{B^{*}}-1\right),\\ L\left(-B^{*}\ln\frac{B}{B^{*}}\right) & =-\frac{\eta B^{*}}{B}-cB^{*}V+\eta +cB^{*}V^{*}\\ & =\eta \left(1-\frac{B^{*}}{B}\right)+cB^{*}V^{*}\left(1-\frac{V}{V^{*}}\right),\\ L\left(-Z^{*}\ln\frac{Z}{Z^{*}}\right) & =-\frac{\gamma Z^{*}}{Z}-\omega IZ^{*}+\gamma +\omega I^{*}Z^{*}\\ & =\gamma \left(1-\frac{Z^{*}}{Z}\right)+\omega I^{*}Z^{*}\left(1-\frac{I}{I^{*}}\right). \end{array}$$

Using (13) and the inequality *x* ≥ ln *x* + 1(*∀x* > 0), we obtain

$$\begin{array}{l} L\left[S^{*}H\left(\frac{S}{S^{*}}\right)+I^{*}H\left(\frac{I}{I^{*}}\right)\right]\\ \leq \lambda -dS-aI-\epsilon IZ+\frac{dS^{*}}{S}\left(S-S^{*}\right)+\epsilon I^{*}Z^{*}\left(\frac{Z}{Z^{*}}-1\right)+\bar{b}S^{*}F\left(V^{*}\right)\left[\frac{F\left(V\right)}{F\left(V^{*}\right)}-\frac{S^{*}}{S}+1-\frac{e^{x}SF\left(V\right)I^{*}}{S^{*}F\left(V^{*}\right)I}\right]\\ \;\;\;\;+\left|e^{x}-1\right|\bar{b}S^{*}F\left(V\right)\\ =-d\frac{{\left(S-S^{*}\right)}^{2}}{S}+\epsilon I^{*}Z^{*}\left(\frac{I}{I^{*}}+\frac{Z}{Z^{*}}-1-\frac{IZ}{I^{*}Z^{*}}\right)+\bar{b}S^{*}F\left(V^{*}\right)\left[\frac{F\left(V\right)}{F\left(V^{*}\right)}+1-\frac{S^{*}}{S}+1-\frac{e^{x}SF\left(V\right)I^{*}}{S^{*}F\left(V^{*}\right)I}-\frac{I}{I^{*}}\right]\\ \;\;\;\;+\left|e^{x}-1\right|\bar{b}S^{*}F\left(V\right)\\ \leq -d\frac{{\left(S-S^{*}\right)}^{2}}{S_{0}}+\epsilon I^{*}Z^{*}\left(\frac{I}{I^{*}}-\ln\frac{I}{I^{*}}-1+\frac{Z}{Z^{*}}-\ln\frac{Z}{Z^{*}}-1-\frac{IZ}{I^{*}Z^{*}}+\ln\frac{IZ}{I^{*}Z^{*}}+1\right)\\ \;\;\;\;+\bar{b}S^{*}F\left(V^{*}\right)\left[\frac{F\left(V\right)}{F\left(V^{*}\right)}-x-\ln\frac{F\left(V\right)}{F\left(V^{*}\right)}+\ln\frac{I}{I^{*}}-\frac{I}{I^{*}}\right]+\left|e^{x}-1\right|\bar{b}S^{*}F^{'}\left(0\right)M_{2}\\ \leq -d\frac{{\left(S-S^{*}\right)}^{2}}{S_{0}}+\epsilon I^{*}Z^{*}\left[H\left(\frac{I}{I^{*}}\right)+H\left(\frac{Z}{Z^{*}}\right)-H\left(\frac{IZ}{I^{*}Z^{*}}\right)\right]+\bar{b}S^{*}F\left(V^{*}\right)H\left[\frac{F\left(V\right)}{F\left(V^{*}\right)}\right]-bS^{*}F\left(V^{*}\right)H\left(\frac{I}{I^{*}}\right)\\ \;\;\;\;+\left|e^{x}-1\right|\bar{b}S^{*}F^{'}\left(0\right)M_{2}+\bar{b}S^{*}F\left(V^{*}\right)x. \end{array}$$

Moreover,

$$\begin{array}{l} L\left[V^{*}H\left(\frac{V}{V^{*}}\right)\right]\\ =mI-rV-qVB+V^{*}\left(-\frac{mI}{V}+r+qB\right)\\ \leq mI-rV-qVB-mI^{*}\left(\ln\frac{I}{I^{*}}-\ln\frac{V}{V^{*}}\right)+qV^{*}B^{*}\left(\frac{B}{B^{*}}-1\right)\\ =mI^{*}\left(\frac{I}{I^{*}}-\ln\frac{I}{I^{*}}-1\right)-rV^{*}\left(\frac{V}{V^{*}}-\ln\frac{V}{V^{*}}-1\right)+qV^{*}B^{*}\left(\frac{B}{B^{*}}-\ln\frac{B}{B^{*}}-1-\frac{VB}{V^{*}B^{*}}+\ln\frac{VB}{V^{*}B^{*}}+1\right)\\ =mI^{*}H\left(\frac{I}{I^{*}}\right)-rV^{*}H\left(\frac{V}{V^{*}}\right)+qV^{*}B^{*}\left[H\left(\frac{B}{B^{*}}\right)-H\left(\frac{VB}{V^{*}B^{*}}\right)\right]. \end{array}$$

Similarly, we get

$$\begin{array}{l} L\left[B^{*}H\left(\frac{B}{B^{*}}\right)\right]\\ =\eta +cBV-\delta B+\eta \left(1-\frac{B^{*}}{B}\right)+cB^{*}V^{*}\left(1-\frac{V}{V^{*}}\right)\\ =-\frac{\eta }{B^{*}}\frac{{\left(B-B^{*}\right)}^{2}}{B}+cB^{*}V^{*}\left(\frac{BV}{B^{*}V^{*}}-\frac{B}{B^{*}}+1-\frac{V}{V^{*}}\right)\\ \leq -\frac{\eta }{B^{*}}\frac{{\left(B-B^{*}\right)}^{2}}{M_{3}}+cB^{*}V^{*}\left(\frac{BV}{B^{*}V^{*}}-\ln\frac{BV}{B^{*}V^{*}}-1-\frac{B}{B^{*}}+\ln\frac{B}{B^{*}}+1-\frac{V}{V^{*}}+\ln\frac{V}{V^{*}}+1\right)\\ =-\frac{\eta }{B^{*}}\frac{{\left(B-B^{*}\right)}^{2}}{M_{3}}+cB^{*}V^{*}\left[H\left(\frac{BV}{B^{*}V^{*}}\right)-H\left(\frac{B}{B^{*}}\right)-H\left(\frac{V}{V^{*}}\right)\right], \end{array}$$

and

$$\begin{array}{ll} L\left[Z^{*}H\left(\frac{Z}{Z^{*}}\right)\right] & \\ =\gamma +\omega IZ-\frac{\gamma +\omega I^{*}Z^{*}}{Z^{*}}Z+\gamma \left(1-\frac{Z^{*}}{Z}\right)+\omega I^{*}Z^{*}\left(1-\frac{I}{I^{*}}\right) & \\ =-\frac{\gamma }{Z^{*}}\frac{{\left(Z-Z^{*}\right)}^{2}}{Z}+\omega I^{*}Z^{*}\left(\frac{IZ}{I^{*}Z^{*}}-\frac{Z}{Z^{*}}+1-\frac{I}{I^{*}}\right) & \\ \leq -\frac{\gamma }{Z^{*}}\frac{{\left(Z-Z^{*}\right)}^{2}}{M_{4}}+\omega I^{*}Z^{*}\left(\frac{IZ}{I^{*}Z^{*}}-\ln\frac{IZ}{I^{*}Z^{*}}-1-\frac{Z}{Z^{*}}+\ln\frac{Z}{Z^{*}}+1-\frac{I}{I^{*}}+\ln\frac{I}{I^{*}}+1\right) & \\ =-\frac{\gamma }{Z^{*}}\frac{{\left(Z-Z^{*}\right)}^{2}}{M_{4}}+\omega I^{*}Z^{*}\left[H\left(\frac{IZ}{I^{*}Z^{*}}\right)-H\left(\frac{Z}{Z^{*}}\right)-H\left(\frac{I}{I^{*}}\right)\right]. & \end{array}$$

Let

$$P_{3}=S^{*}H\left(\frac{S}{S^{*}}\right)+I^{*}H\left(\frac{I}{I^{*}}\right)+\frac{\bar{b}S^{*}F\left(V^{*}\right)}{mI^{*}}V^{*}H\left(\frac{V}{V^{*}}\right)+\frac{q\bar{b}S^{*}F\left(V^{*}\right)}{cmI^{*}}H\left(\frac{B}{B^{*}}\right)+\frac{\epsilon }{\omega }Z^{*}H\left(\frac{Z}{Z^{*}}\right).$$

Applying It$\hat{o}$’s formula, we have

$$\begin{array}{l} LP_{3}\leq -d\frac{{\left(S-S^{*}\right)}^{2}}{S_{0}}-\frac{\eta q\bar{b}S^{*}F\left(V^{*}\right)}{cmI^{*}B^{*}}\frac{{\left(B-B^{*}\right)}^{2}}{M_{3}}-\frac{\epsilon \gamma }{\omega Z^{*}}\frac{{\left(Z-Z^{*}\right)}^{2}}{M_{4}}+\left|e^{x}-1\right|\bar{b}S^{*}F^{'}\left(0\right)M_{2}+\bar{b}S^{*}F\left(V^{*}\right)x\\ \;\;\;\;\;\;\;\;\;\;\;+\bar{b}S^{*}F\left(V^{*}\right)H\left[\frac{F\left(V\right)}{F\left(V^{*}\right)}\right]-\frac{\bar{b}S^{*}F\left(V^{*}\right)}{mI^{*}}\left(rV^{*}+qB^{*}V^{*}\right)H\left(\frac{V}{V^{*}}\right). \end{array}$$

From equation (13), it can be derived that

$$\begin{array}{l} \bar{b}S^{*}F\left(V^{*}\right)H\left[\frac{F\left(V\right)}{F\left(V^{*}\right)}\right]-\frac{\bar{b}S^{*}F\left(V^{*}\right)}{mI^{*}}\left(rV^{*}+qB^{*}V^{*}\right)H\left(\frac{V}{V^{*}}\right)\\ =\bar{b}S^{*}F\left(V^{*}\right)\left[\frac{F\left(V\right)}{F\left(V^{*}\right)}-\ln\frac{F\left(V\right)}{F\left(V^{*}\right)}-\frac{V}{V^{*}}+\ln\frac{V}{V^{*}}\right]\\ =\bar{b}S^{*}F\left(V^{*}\right)\left[\frac{F\left(V\right)}{F\left(V^{*}\right)}-\frac{V}{V^{*}}+\ln\frac{VF\left(V^{*}\right)}{V^{*}F\left(V\right)}\right]\\ \leq \bar{b}S^{*}F\left(V^{*}\right)\left[\frac{F\left(V\right)}{F\left(V^{*}\right)}-\frac{V}{V^{*}}+\frac{VF\left(V^{*}\right)}{V^{*}F\left(V\right)}-1\right]\\ =\bar{b}S^{*}F\left(V^{*}\right)\left[\frac{F\left(V\right)-F\left(V^{*}\right)}{F\left(V^{*}\right)}-\frac{V\left(F\left(V\right)-F\left(V^{*}\right)\right)}{V^{*}F\left(V\right)}\right]\\ =\bar{b}S^{*}F\left(V^{*}\right)\left[F\left(V\right)-F\left(V^{*}\right)\right]\frac{V^{*}F\left(V\right)-VF\left(V^{*}\right)}{F\left(V^{*}\right)V^{*}F\left(V\right)}. \end{array}$$

By the Lagrange median theorem, it follows that

$$F\left(V\right)-F\left(V^{*}\right)=F^{\prime }\left(\xi_{1}\right)\left(V-V^{*}\right),$$

and

$$\frac{V^{*}F\left(V\right)-VF\left(V^{*}\right)}{V^{*}V}=\frac{F\left(V\right)}{V}-\frac{F\left(V^{*}\right)}{V^{*}}={\left({\left(\frac{F\left(V\right)}{V}\right)}^{\prime }\right|}_{V=\xi_{2}}\left(V-V^{*}\right),$$

where *ξ*_1_, *ξ*_2_ are the values between *V*∗ and *V*.

Moreover, from conditions ($A_{2}$) and ($A_{3}$), we obtain

$$F^{\prime }\left(\xi_{1}\right)>0,\;{\left({\left(\frac{F\left(V\right)}{V}\right)}^{\prime }\right|}_{V=\xi_{2}}\leq 0.$$

Therefore, we have

$$\begin{array}{l} \left[F\left(V\right)-F\left(V^{*}\right)\right]\frac{V^{*}F\left(V\right)-VF\left(V^{*}\right)}{F\left(V^{*}\right)V^{*}F\left(V\right)}\\ =F^{'}\left(\xi_{1}\right)\left(V-V^{*}\right)\cdot \frac{V}{F\left(V^{*}\right)F\left(V\right)}{\left({\left(\frac{F\left(V\right)}{V}\right)}^{'}\right|}_{V=\xi_{2}}\left(V-V^{*}\right)\\ =F^{'}\left(\xi_{1}\right){\left({\left(\frac{F\left(V\right)}{V}\right)}^{'}\right|}_{V=\xi_{2}}\frac{V}{F\left(V^{*}\right)F\left(V\right)}{\left(V-V^{*}\right)}^{2}\leq 0. \end{array}$$

Thus

$$LP_{3}\leq -d\frac{{\left(S-S^{*}\right)}^{2}}{S_{0}}-\frac{\eta q\bar{b}S^{*}F\left(V^{*}\right)}{cmI^{*}B^{*}}\frac{{\left(B-B^{*}\right)}^{2}}{M_{3}}-\frac{\epsilon \gamma }{\omega Z^{*}}\frac{{\left(Z-Z^{*}\right)}^{2}}{M_{4}}+\left|e^{x}-1\right|\bar{b}S^{*}F^{\prime }\left(0\right)M_{2}+\bar{b}S^{*}F\left(V^{*}\right)\left(\ln\;\bar{b}-\ln\;b\right).$$

In view of system (5), one gets

$$L\left(S+I+\frac{\epsilon }{\omega }Z\right)=\lambda -dS-aI+\frac{\epsilon \gamma }{\omega }-\frac{\epsilon \mu }{\omega }Z=-d\left(S-S^{*}\right)-a\left(I-I^{*}\right)-\frac{\epsilon \mu }{\omega }\left(Z-Z^{*}\right).$$

Applying It$\hat{o}$’s formula and Young's inequality, we have

$$\begin{array}{l} \;\\ L\left[\frac{1}{2}{\left(S-S^{*}+I-I^{*}+\frac{\epsilon }{\omega }\left(Z-Z^{*}\right)\right)}^{2}\right]\\ =-d{\left(S-S^{*}\right)}^{2}-a{\left(I-I^{*}\right)}^{2}-\frac{\epsilon^{2}\mu }{\omega^{2}}{\left(Z-Z^{*}\right)}^{2}-\left(a+d\right)\left(I-I^{*}\right)\left(S-S^{*}\right)-\frac{\epsilon \left(\mu +a\right)}{\omega }\left(I-I^{*}\right)\left(Z-Z^{*}\right)-\frac{\epsilon \left(\mu +d\right)}{\omega }\left(S-S^{*}\right)\left(Z-Z^{*}\right)\\ \leq -d{\left(S-S^{*}\right)}^{2}-a{\left(I-I^{*}\right)}^{2}-\frac{\epsilon^{2}\mu }{\omega^{2}}{\left(Z-Z^{*}\right)}^{2}+\frac{a}{4}{\left(I-I^{*}\right)}^{2}+\frac{{\left(a+d\right)}^{2}}{a}{\left(S-S^{*}\right)}^{2}+\frac{a}{4}{\left(I-I^{*}\right)}^{2}\\ \;\;\;\;+\frac{\epsilon^{2}{\left(\mu +a\right)}^{2}}{a\omega^{2}}{\left(Z-Z^{*}\right)}^{2}+\frac{d}{2}{\left(S-S^{*}\right)}^{2}+\frac{1}{2d}\frac{\epsilon^{2}{\left(\mu +d\right)}^{2}}{\omega^{2}}{\left(Z-Z^{*}\right)}^{2}\\ =-\frac{a}{2}{\left(I-I^{*}\right)}^{2}+E_{1}{\left(S-S^{*}\right)}^{2}+E_{2}{\left(Z-Z^{*}\right)}^{2}, \end{array}$$

where the forms of *E*_1_ and *E*_2_ are as shown in equation (9). Through similar calculations, we have

$$L\left(V+\frac{q}{c}B\right)=m\left(I-I^{*}\right)-r\left(V-V^{*}\right)-\frac{q\delta }{c}\left(B-B^{*}\right),$$

and

$$\begin{array}{l} \;\\ L\left[\frac{1}{2}{\left(V-V^{*}+\frac{q}{c}\left(B-B^{*}\right)\right)}^{2}\right]\\ =-r{\left(V-V^{*}\right)}^{2}-\frac{q^{2}\delta }{c}{\left(B-B^{*}\right)}^{2}+m\left(I-I^{*}\right)\left(V-V^{*}\right)-\frac{qm}{c}\left(I-I^{*}\right)\left(B-B^{*}\right)-\frac{q\left(\delta +r\right)}{c}\left(V-V^{*}\right)\left(B-B^{*}\right)\\ \leq -r{\left(V-V^{*}\right)}^{2}-\frac{q^{2}\delta }{c}\left(B-B^{*}\right)+\frac{r}{4}\left(V-V^{*}\right)+\frac{m^{2}}{r}{\left(I-I^{*}\right)}^{2}+\frac{m^{2}}{2r}{\left(I-I^{*}\right)}^{2}+\frac{r}{2m^{2}}\cdot \frac{q^{2}m^{2}}{c^{2}}{\left(B-B^{*}\right)}^{2}\\ \;\;\;\;+\frac{r}{4}{\left(V-V^{*}\right)}^{2}+\frac{q^{2}{\left(\delta +r\right)}^{2}}{rc^{2}}{\left(B-B^{*}\right)}^{2}\\ =-\frac{r}{2}{\left(V-V^{*}\right)}^{2}+\frac{3m^{2}}{2r}{\left(I-I^{*}\right)}^{2}+g_{1}{\left(B-B^{*}\right)}^{2}, \end{array}$$

where $g_{1}=\frac{q^{2}r}{2c^{2}}+\frac{q^{2}\left(\delta^{2}+r^{2}+r\delta \right)}{rc^{2}}$. Let

$$P_{4}=\frac{1}{2}{\left[S-S^{*}+I-I^{*}+\frac{\epsilon }{\omega }\left(Z-Z^{*}\right)\right]}^{2}+\frac{ar}{6m^{2}}\left[\frac{1}{2}{\left(V-V^{*}+\frac{q}{c}\left(B-B^{*}\right)\right)}^{2}\right],$$

then we get

$$LP_{4}\leq -\frac{a}{4}{\left(I-I^{*}\right)}^{2}-\frac{ar^{2}}{12m^{2}}{\left(V-V^{*}\right)}^{2}+E_{1}{\left(S-S^{*}\right)}^{2}+E_{2}{\left(Z-Z^{*}\right)}^{2}+E_{3}{\left(B-B^{*}\right)}^{2},$$

where. $E_{3}=\frac{arg_{1}}{6m^{2}}.$

Define

$$\tilde{P}=P_{3}+J_{2}P_{4},$$

where $J_{2}=\min\left\{\frac{d}{2S_{0}E_{1}},\;\frac{\epsilon \gamma }{2\omega Z^{*}M_{4}E_{2}},\;\frac{\eta q\bar{b}S^{*}F\left(V^{*}\right)}{2cmI^{*}B^{*}M_{3}E_{3}}\right\}$. Then

(14) $$L\tilde{P}\leq -\frac{d}{2S_{0}}{\left(S-S^{*}\right)}^{2}-\frac{aJ_{2}}{4}{\left(I-I^{*}\right)}^{2}-\frac{ar^{2}J_{2}}{12m^{2}}{\left(V-V^{*}\right)}^{2}-\frac{\eta q\bar{b}S^{*}F\left(V^{*}\right)}{2cmI^{*}B^{*}M_{3}}{\left(B-B^{*}\right)}^{2}-\frac{\epsilon \gamma }{2\omega Z^{*}M_{4}}{\left(Z-Z^{*}\right)}^{2}+\left|e^{x}-1\right|\bar{b}S^{*}F^{'}\left(0\right)M_{2}+\bar{b}S^{*}F\left(V^{*}\right)\left(\ln\;\bar{b}-\ln\;b\right)\leq -m_{2}\left[{\left(S-S^{*}\right)}^{2}+{\left(I-I^{*}\right)}^{2}+{\left(V-V^{*}\right)}^{2}+{\left(B-B^{*}\right)}^{2}+{\left(Z-Z^{*}\right)}^{2}\right]+\left|e^{x}-1\right|\bar{b}S^{*}F^{'}\left(0\right)M_{2}+\bar{b}S^{*}F\left(V^{*}\right)\left(\ln\;\bar{b}-\ln\;b\right),$$

and

(15) $$m_{2}=\min\left\{\frac{d}{2S_{0}},\;\frac{aJ_{2}}{4},\;\frac{ar^{2}J_{2}}{12m^{2}},\;\frac{\eta q\bar{b}S^{*}F\left(V^{*}\right)}{2cmI^{*}B^{*}M_{3}},\;\frac{\epsilon \gamma }{2\omega Z^{*}M_{4}}\right\}.$$

After a calculation similar to the proof of Theorem 3.1, it is derived that

$$\begin{array}{l} m_{2}{\mathrm{limsup}}_{t\to \infty }E\left[\frac{1}{t}\int_{0}^{t}\left({\left(S\left(\tau \right)-S^{*}\right)}^{2}+{\left(I\left(\tau \right)-I^{*}\right)}^{2}+{\left(V\left(\tau \right)-V^{*}\right)}^{2}+{\left(B\left(\tau \right)-B^{*}\right)}^{2}+{\left(Z\left(\tau \right)-Z^{*}\right)}^{2}\right)d\tau \right]\\ \leq \bar{b}S^{*}F^{'}\left(0\right)M_{2}{\mathrm{limsup}}_{t\to \infty }E\left[\frac{1}{t}\int_{0}^{t}\left|e^{x\left(\tau \right)}-1\right|d\tau \right]+\bar{b}S^{*}F\left(V^{*}\right){\mathrm{limsup}}_{t\to \infty }E\left[\frac{1}{t}\int_{0}^{t}x\left(\tau \right)d\tau \right]\\ \leq S^{*}F^{'}\left(0\right)M_{2}\bar{b}{\left(e^{\frac{\sigma^{2}}{\rho }}-2e^{\frac{\sigma^{2}}{4\rho }}+1\right)}^{\frac{1}{2}}+\bar{b}S^{*}F\left(V^{*}\right){\mathrm{limsup}}_{t\to \infty }E\left[\frac{1}{t}\int_{0}^{t}\left(\ln\;b\left(\tau \right)-\ln\;\bar{b}\right)d\tau \right]. \end{array}$$

Since

$$\lim_{t\to \infty }\frac{1}{t}\int_{0}^{t}\left(\ln\;b\left(\tau \right)-\ln\;\bar{b}\right)\;d\tau =\lim_{t\to \infty }\frac{1}{t}\int_{0}^{t}\ln\;b\left(\tau \right)\;d\tau -\ln\;\bar{b}=0,\text{ a.s. }$$

Then we get

$$\begin{array}{ll} & {\mathrm{lim sup}}_{t\to \infty }E\left[\frac{1}{t}\int_{0}^{t}\left({\left(S\left(\tau \right)-S^{*}\right)}^{2}+{\left(I\left(\tau \right)-I^{*}\right)}^{2}+{\left(V\left(\tau \right)-V^{*}\right)}^{2}+{\left(B\left(\tau \right)-B^{*}\right)}^{2}+{\left(Z\left(\tau \right)-Z^{*}\right)}^{2}\right)d\tau \right]\\ & \;\;\leq \frac{S^{*}F^{\prime }\left(0\right)M_{2}\bar{b}}{m_{2}}{\left(e^{\frac{\sigma^{2}}{\rho }}-2e^{\frac{\sigma^{2}}{4\rho }}+1\right)}^{\frac{1}{2}}. \end{array}$$

Thus, the proof is complete.

From Theorem 3.2, if *R*_0_ > 1, we conclude that the solution of system (5) fluctuates around the infection steady state *Q*∗. In particular, when the noise intensity is zero (*σ* = 0), inequality (14) can be simplified as

$$L\tilde{P}\leq -m_{2}\left[{\left(S-S^{*}\right)}^{2}+{\left(I-I^{*}\right)}^{2}+{\left(V-V^{*}\right)}^{2}+{\left(B-B^{*}\right)}^{2}+{\left(Z-Z^{*}\right)}^{2}\right].$$

The right hand term above is in the form of a negative-definite quadratic form. By the Lyapunov method, we obtain the following conclusion.

Corollary 3.2 *Under conditions* $\left(A_{2}\right)$ *and* $\left(A_{3}\right)$*, if* *R*_0_ > 1*, the infection steady state* $Q^{*}\left(S^{*},I^{*},V^{*},B^{*},Z^{*}\right)$ *of system* (2) *is globally asymptotically stable on the invariant set* Γ.

Based on Theorem 3.1, Theorem 3.2, as well as Corollary 3.1, Corollary 3.2, we find that the concept of the dynamic behavior of the stochastic model at steady state generalizes the global asymptotic stability of the corresponding deterministic model.

## 4 Stationary distribution and extinction

In the deterministic model, we focus on the global asymptotic stability of the steady states. In contrast, for the stochastic CHIKV model, where the concept of steady state no longer exists, our attention shifts to the persistence of target cells, infected cells, virions, B-cells, and CTLs, which can be characterized by the stationary distribution. Given that CHIKV-induced disease poses a major threat to public health and has long term impacts on infected individuals, studying the conditions for viral extinction is particularly critical. Therefore, in this section, we examine the conditions for viral persistence and extinction.

Define a stochastic reproduction number

$$R_{0}^{s}=\frac{\bar{b}m\lambda \mu \delta e^{\frac{\sigma^{2}}{16\rho }}F^{\prime }\left(0\right)}{d\left(a\mu +\epsilon \gamma \right)\left(r\delta +q\eta \right)}.$$

Theorem 4.1 *Under conditions (*$A_{2}$*) and (*$A_{3}$*), if* $R_{0}^{s}>1$*, then system* (5) *has at least one stationary distribution* *π*(⋅) *on* Γ.

The proof of Theorem 4.1 is given in Appendix C. According to Theorem 4.1, when $R_{0}^{s}>1$, system (5) has at least one stationary distribution, indicating that the target cells, infected cells, free CHIKV particles, B-cells, and CTLs can be maintained at certain levels within the host. By comparing *R*_0_ with $R_{0}^{s}$, it is evident that $R_{0}^{s}\geq R_{0}$, with equality only occurring when *σ* = 0. Therefore, the existence of the stationary distribution in the stochastic model is a generalization of the stability of the infection steady state in the corresponding deterministic model. Thus $R_{0}^{s}>1$ can be regarded as a unified condition for the persistence of CHIKV in both the deterministic and stochastic models.

Define

$$R_{0}^{e}=\sqrt{R_{0}}+\frac{\left(r\delta +q\eta \right)\sqrt{R_{0}}{\left(e^{\frac{\sigma^{2}}{\rho }}-2e^{\frac{\sigma^{2}}{4\rho }}+1\right)}^{\frac{1}{2}}}{\delta \min\left\{a+\frac{\epsilon \gamma }{\mu },r+\frac{q\eta }{\delta }\right\}}.$$

Theorem 4.2 *Let* *X*(*t*) *be the solution of system* (5) *with any initial value* *X*_0_ ∈ Γ*. The solution* *X*(*t*) *of system* (5) *satisfies*

$${\mathrm{lim sup}}_{t\to \infty }\frac{1}{t}\ln\left(\frac{\mu }{a\mu +\epsilon \gamma }I+\frac{\sqrt{R_{0}}}{m}V\right)\leq \min\left\{a+\frac{\epsilon \gamma }{\mu },r+\frac{q\eta }{\delta }\right\}\left(R_{0}^{e}-1\right),\;\;\text{a.s. }$$

If $R_{0}^{e}<1$, *the infected cells and CHIKV particles will become exponentially extinct in a long term*; *that is*,

$$\lim_{t\to \infty }I\left(t\right)=\lim_{t\to \infty }V\left(t\right)=0,\;\text{ a.s. }$$

The proof of Theorem 4.2 is given in Appendix D.

## 5 Probability density functions

In Section 4, we verify the existence of the stationary distribution of system (5). To further explore the property of the stationary distribution, we provide an approximate expression for the probability density function of system (5) in the vicinity of the quasi-steady state.

We analyze the stochastic system (5) around the quasi-steady state $\tilde{Q^{*}}\left(S^{*},I^{*},V^{*},B^{*},Z^{*},0\right)$. First, we perform an equivalent transformation of the system. Let $Y={\left(y_{1},y_{2},y_{3},y_{4},y_{5},y_{6}\right)}^{\text{T}}={\left(S-S^{*},I-I^{*},V-V^{*},B-B^{*},Z-Z^{*},x\right)}^{\text{T}}$, and we obtain the linearized system corresponding to system (5) as follows:

(16) $$\begin{array}{l} \left\{\begin{array}{l} dy_{1}=\left(-a_{11}y_{1}-a_{13}y_{3}-a_{16}y_{6}\right)dt,\;\\ dy_{2}=\left(a_{21}y_{1}-a_{22}y_{2}+a_{13}y_{3}-a_{25}y_{5}+a_{16}y_{6}\right)dt,\;\\ dy_{3}=\left(a_{32}y_{2}-a_{33}y_{3}-a_{34}y_{4}\right)dt,\;\\ dy_{4}=\left(a_{43}y_{3}-a_{44}y_{4}\right)dt,\;\\ dy_{5}=\left(a_{52}y_{2}-a_{55}y_{5}\right)dt,\;\\ dy_{6}=-\rho y_{6}dt+\sigma \;dB\left(t\right),\; \end{array}\right) \end{array}$$

where

$$\begin{array}{ll} & a_{11}=d+\bar{b}F\left(V^{*}\right),\;a_{13}=\bar{b}S^{*}F^{\prime }\left(V^{*}\right),\;a_{16}=\bar{b}S^{*}F\left(V^{*}\right),\;a_{21}=\bar{b}F\left(V^{*}\right),\;a_{22}=a+\epsilon Z^{*},\;a_{25}=\epsilon I^{*},\;a_{32}=m,\\ & a_{33}=r+qB^{*},\;a_{34}=qV^{*},a_{43}=cB^{*},\;a_{44}=\delta -cV^{*}=\frac{\eta }{B^{*}},\;a_{52}=\omega Z^{*},\;a_{55}=\mu -\omega I^{*}=\frac{\gamma }{Z^{*}}. \end{array}$$

System (16) can equally be rewritten as

$$dY\left(t\right)=A_{0}Y\left(t\right)+H_{0}dB\left(t\right),$$

where

$$\begin{array}{l} A_{0}=\left(\begin{array}{llllll} -a_{11} & 0 & -a_{13} & 0 & 0 & -a_{16}\\ a_{21} & -a_{22} & a_{13} & 0 & -a_{25} & a_{16}\\ 0 & a_{32} & -a_{33} & -a_{34} & 0 & 0\\ 0 & 0 & a_{43} & -a_{44} & 0 & 0\\ 0 & a_{52} & 0 & 0 & -a_{55} & 0\\ 0 & 0 & 0 & 0 & 0 & -\rho \end{array}\right), \end{array}$$

and *H*_0_ = diag(0, 0, 0, 0, 0, *σ*).

Theorem 5.1 *Under conditions* $\left(A_{2}\right)$ *and* $\left(A_{3}\right)$*, if* *b*_4_ ≠ 0*,* *b*_8_ ≠ 0 *and* *R*_0_ > 1*, the stationary solution* *X*(*t*) *of system* (5) *approximately obeys a normal distribution and the probability density function* Φ(*X*) *is expressed as follows:*

$$\Phi \left(X\right)={\left(2\pi \right)}^{-3}|\Sigma_{0}{|}^{-\frac{1}{2}}e^{-\frac{1}{2}\left(X-\tilde{Q^{*}}\right)\Sigma_{0}^{-1}{\left(X-\tilde{Q^{*}}\right)}^{\text{T}}},$$

*where* Σ_0_ *is a positive definite matrix and*

$$\Sigma_{0}={\left(I_{7}I_{6}I_{5}I_{4}I_{3}I_{2}I_{1}\right)}^{-1}\left[k^{2}T_{0}^{-1}\Xi {\left(T_{0}^{-1}\right)}^{\text{T}}\right]{\left({\left(I_{7}I_{6}I_{5}I_{4}I_{3}I_{2}I_{1}\right)}^{-1}\right)}^{\text{T}}.$$

*Here,* Ξ *can be found in equation* (*E*.3)*,* *I*_*i*_ (*i* = 1, 2, …, 6)*,* *k* *and* *T*_0_ *are provided in* Appendix E.

The proof of Theorem 5.1 is given in Appendix E.

## 6 Uncertainty and sensitivity analysis

To quantitatively assess how parameter uncertainty affects each population of stochastic model (5) and their corresponding deterministic model (2), we adopt a sensitivity analysis method based on Latin Hypercube Sampling (LHS) and Partial Rank Correlation Coefficient (PRCC) (Blower & Dowlatabadi, 1994; Marino et al., 2008; McKay et al., 1979). In our analysis, we consider system (5) with a bilinear incidence rate *F*(*V*) = *V*, and the equation can be written as

(17) $$\left\{\begin{array}{l} dS=\left[\lambda -dS-\bar{b}e^{x}SV\right]\;dt,\\ dI=\left[\bar{b}e^{x}SV-aI-\epsilon IZ\right]\;dt,\\ dV=\left[mI-rV-qVB\right]\;dt,\\ dB=\left[\eta +cBV-\delta B\right]\;dt,\\ dZ=\left[\gamma +\omega IZ-\mu Z\right]\;dt,\\ dx=-\rho x\;dt+\sigma \;\mathrm{dB}\left(t\right). \end{array}\right)$$

According to the results of Section 4, we obtain the stochastic reproduction number

$$R_{0}^{s}=\frac{\bar{b}m\lambda \mu \delta e^{\frac{\sigma^{2}}{16\rho }}}{d\left(a\mu +\epsilon \gamma \right)\left(r\delta +q\eta \right)}.$$

We define baseline values and the reasonable range of the sixteen parameters in system (17) (see Table 2). Assume that all parameters follow uniform distribution. Next, we primarily perform sensitivity analysis from two aspects: one is the analysis of each population, and the other is the analysis of the value of the stochastic reproduction number.

**Table 2:** Parameter values.

Parameter	Baseline	Range	Unit	Source
*λ*	1.826	[1, 3]	cells μl day^−1^	Wang and Liu (2017)
*d*	0.7979	[0.4, 1.2]	day^−1^	(Patel et al., 2017; Wang & Liu, 2017)
*b*	0.9	[0.1, 1.5]	μl virion^−1^ day^−1^	Wang and Liu (2017)
*a*	0.5	[0.2, 0.7]	day^−1^	(Patel et al., 2017; Wang & Liu, 2017)
*ϵ*	0.4441	[0.3, 0.6]	μl cell^−1^ day^−1^	Alade et al. (2023)
*m*	2.2	[1.5, 3]	virions/cell	Cavalcanti et al. (2022)
*r*	0.4	[0.2, 0.6]	day^−1^	Wang and Liu (2017)
*q*	0.55	[0.4, 0.7]	μl cell^−1^ day^−1^	Cavalcanti et al. (2022)
*η*	1.402	[1, 2]	day^−1^	Wang and Liu (2017)
*c*	0.9	[0.5, 1.5]	μl cell^−1^ day^−1^	Cavalcanti et al. (2022)
*δ*	1.251	[0.6, 1.8]	day^−1^	Assumed
*γ*	0.5	[0.2, 0.7]	day^−1^	Alade et al. (2023)
*ω*	0.5	[0.2, 0.7]	μl cell^−1^ day^−1^	Alade et al. (2023)
*μ*	1.35	[0.6, 2]	day^−1^	Alade et al. (2023)
*ρ*	4	[0.1, 6]	–	Assumed
*σ*	0.7	[0.1, 6]	–	Assumed

### 6.1 Analysis of each population

**Fig. 2:**
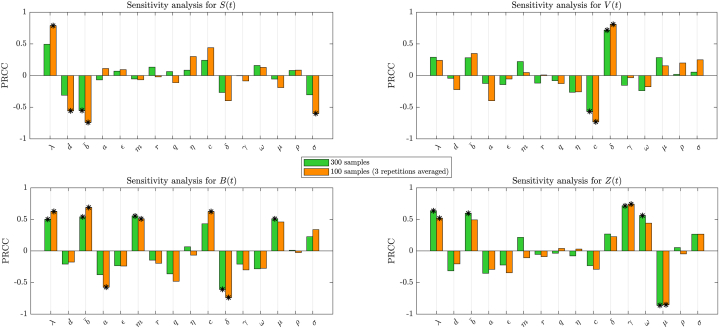
Sensitivity analysis of *S*, *V*, *B*, and *Z* of system (17). The asterisk symbol (∗) indicates the key parameters (p < 0.01 and $\left|\text{PRCC}\right|>0.5$).

Firstly, we apply the LHS method with two sampling strategies: 300 samples and 100 samples. The 300 parameter samples are simulated once, while another set of 100 parameter samples is replicated three times and then averaged, producing 100 sample means (Marino et al., 2008). We calculate the PRCC values and their corresponding p-values for each parameter with respect to each population. The output results are shown in Fig. 2, the symbol (∗) indicates the key parameters (p < 0.01 and $\left|\text{PRCC}\right|>0.5$).

As shown in Fig. 2, the sensitivity analysis of model parameters demonstrates how key parameters impact each population. For target cell population (*S*), *λ* exhibits a significant positive effect, while $\bar{b}$, *d*, and *σ* show strong negative correlations. CHIKV particles (*V*) are negatively effected by *c*, whereas *δ* exerts the opposite effect. For B-cell population (*B*), key parameters $\bar{b}$, *λ*, and *m* contribute positively, whereas *δ* and *a* display negative correlations. For CTL population (*Z*), *γ*, *λ*, and $\bar{b}$ are key positive effects, while *μ* has a negative effect. Overall, *λ* and $\bar{b}$ are the most influential parameters effecting target cell, B-cell and CTL populations.

**Fig. 3:**
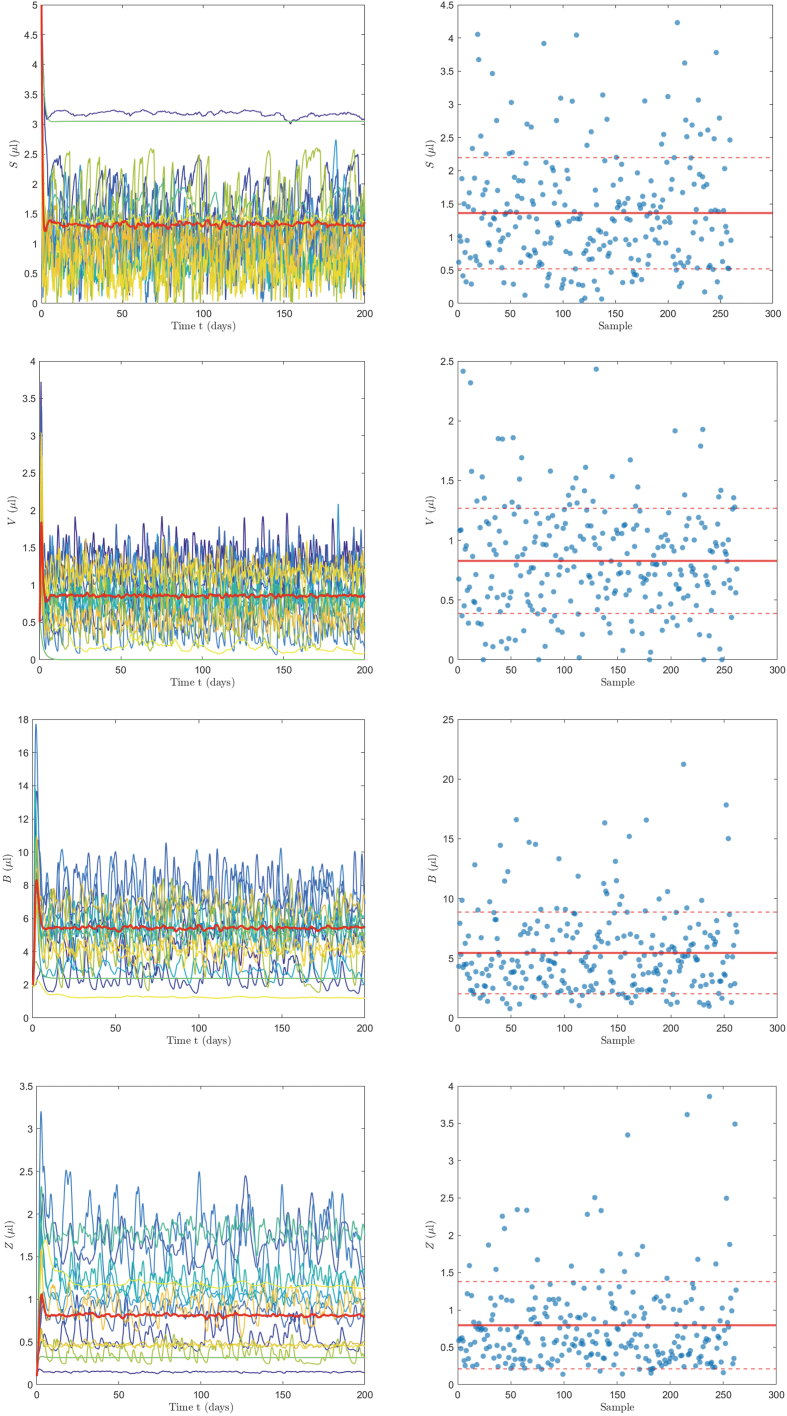
Simulation results of LHS (300 runs) with 15 randomly selected trajectories displayed. Left panel shows the dynamics of *S*, *V*, *B*, and *Z*, with the red line representing the trajectory based on the baseline. Right panel shows the distribution of *S*, *V*, *B*, and *Z* at *t* = 200 days (mean in red, ±1 standard deviation).

Notably, most key parameters show consistent sensitivity under both 300 and 100 sampling repetitions, confirming the robustness of the analysis. Compared with 300 samples, a reduced approach using only 100 samples repeated three times still successfully identified significant effectsłsuch as those of *d* and *σ* on target cell population, and *a* and *c* on B-cell population. This suggests that averaging over repeated simulations with smaller sample sizes remains effective in capturing essential dynamical features, offering a computationally efficient alternative for parameter sensitivity analysis (Marino et al., 2008).Fig. 3 shows the simulation results with a sample size of 300. The left panel of Fig. 3 presents the trend of sample concentration changes over time for each population. The simulation results indicate that, approximately two weeks after infection, the fluctuations in the concentration of each population tend to stabilize, with this timescale corresponding closely to the acute phase of CHIKV infection (Bartholomeeusen et al., 2023; Gonsalves et al., 2025). The right panel of Fig. 3 shows the distribution of the concentrations of each population at *t* = 200 days, illustrating the variation in the steady state values of each population under different parameter combinations. It can be observed that at *t* = 200, most of the sample values are clustered within one standard deviation of the mean.

### 6.2 Analysis of the stochastic reproduction number

We conduct parameter sensitivity analysis on both stochastic reproduction number $R_{0}^{s}$ and the basic reproduction number *R*_0_. To assess the impact of the reversion rate and noise intensity on the stochastic reproduction number, we establish six distinct scenarios: (i) low reversion rate and low noise intensity (the baseline value (*ρ*, *σ*)=(2, 0.5)); (ii) high reversion rate and low noise intensity (the baseline value (*ρ*, *σ*)=(4, 0.5)); (iii) low reversion rate and high noise intensity (the baseline value (*ρ*, *σ*)=(2, 4)); (iv) high reversion rate and high noise intensity (the baseline value (*ρ*, *σ*)=(4, 4)); (v) various disturbance level (both parameters (*ρ*, *σ*) are within the range (0.1, 6)); (vi) absence of disturbance. For cases (i) to (iv), we assume variation between 20 % and 200 % of the selected baseline values.

In the uncertainty analysis, we conduct five repeated experiments, each obtaining 1000 samples. The results show that the proportions of $R_{0}^{s}>1$ in cases (i) and (ii) are 95.90 % and 95.88 %, respectively. In cases (iii) and (iv), these proportions increase to 98.74 % and 97.58 %, respectively. In case (v), 95.76 % of the parameter combinations satisfy $R_{0}^{s}>1$. In case (vi), 95.76 % of the parameter combinations satisfy *R*_0_ > 1. It is evident that when the noise intensity in the stochastic model is low, the probability of $R_{0}^{s}>1$ is approximately equal to that of *R*_0_ > 1 in the deterministic model, ranging between 95 % and 96 %. However, when the noise intensity in the stochastic model is high, the probability of $R_{0}^{s}>1$ increases significantly by approximately two percentage points.

**Fig. 4:**
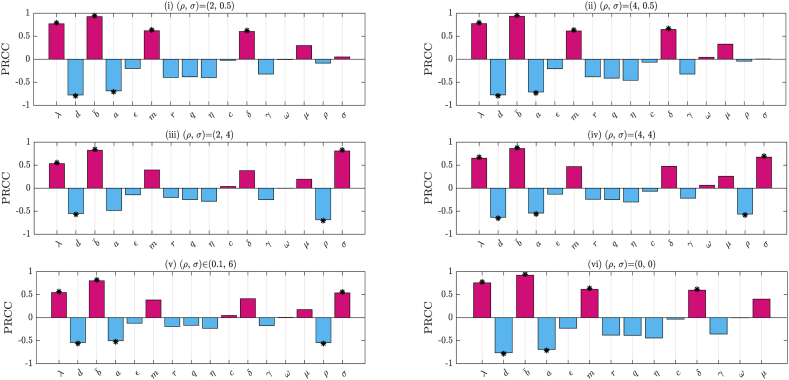
Identification of key parameters influencing the stochastic reproduction number $R_{0}^{s}$ and basic reproduction number *R*_0_. The asterisk symbol (∗) indicates the key parameters (p < 0.01 and $\left|\text{PRCC}\right|>0.5$).

Next, we randomly generate 1000 samples from uniform distributions of relevant parameters. The PRCC values of $R_{0}^{s}$ and *R*_0_ under different parameter conditions are shown in Fig. 4. The results of cases (i) and (ii) show that when the noise intensity is low, *ρ* and *σ* have no significant impact on $R_{0}^{s}$. Parameters $\bar{b}$, *λ*, *m*, and *δ* have significant positive effects on $R_{0}^{s}$, while *d* and *a* have significant negative effects. In contrast, case (iii) and (iv) reveal that when *σ* is high, $\bar{b}$, *σ*, and *λ* all exhibit significant positive effects on $R_{0}^{s}$, while *ρ* and *d* show significant negative effects. Case (v) shows the results under various combinations of *ρ* and *σ*, consistent with the performance in case (iv). For case (vi), we found that in the absence of stochastic factors, the parameters that significantly influence *R*_0_ remain consistent with case (i) and (ii).

In summary, the following conclusions can be drawn: (i) regardless of the levels of *ρ* and *σ*, *λ* and $\bar{b}$ consistently exhibit significant positive effects on the stochastic reproduction number $R_{0}^{s}$, while *d* consistently shows significant negative effects. (ii) when *σ* is at a low level, the influence of both *ρ* and *σ* on $R_{0}^{s}$ is negligible. In contrast, under high *σ* level, both *ρ* and *σ* have a substantial impact on $R_{0}^{s}$. These findings indicate high noise intensity significantly enhances the influence of stochasticity on $R_{0}^{s}$ relative to low noise intensity.

## 7 Numerical simulations

In this section, we describe numerical simulations performed to illustrate the dynamics of the CHIKV model (17). We concentrate on three aspects: (i) the impact of noise intensity *σ* on the number of target cells, infected cells, CHIKV particles, B-cells, and CTLs; (ii) the combined influence of the noise intensity *σ*, the reversion rate *ρ* and the average incidence rate $\bar{b}$ on the two critical conditions $R_{0}^{s}$ and $R_{0}^{e}$; and (iii) the effect of the average incidence rate $\bar{b}$ on the dynamic behavior of the model.

Using Milstein's higher-order method (Higham, 2001) to discretize system (17), the following equations can be obtained:

$$\left\{\begin{array}{l} S_{j+1}=S_{j}+\left(\lambda -dS_{j}-\bar{b}e^{x_{j}}S_{j}V_{j}\right)\Delta t,\;\\ I_{j+1}\;=I_{j}+\left(\bar{b}e^{x_{j}}S_{j}V_{j}-aI_{j}-\epsilon I_{j}Z_{j}\right)\Delta t,\\ V_{j+1}\;=V_{j}+\left(mI_{j}-rV_{j}-qV_{j}B_{j}\right)\Delta t,\\ B_{j+1}=B_{j}+\left(\eta +cB_{j}V_{j}-\delta B_{j}\right)\Delta t,\;\\ Z_{j+1}\;=Z_{j}+\left(\gamma +\omega I_{j}Z_{j}-\mu Z_{j}\right)\Delta t,\\ x_{j+1}\;=x_{j}-\rho x_{j}\Delta t+\sigma \zeta_{j}\sqrt{\Delta t}+\frac{\sigma^{2}}{2}\left(\zeta_{j}^{2}-1\right)\Delta t, \end{array}\right)$$

where Δ*t* indicates the time increments and *ζ*_*j*_ represents the *j*-th iteration of the Brownian motion.

Example 1 (**Stationary Distribution and Probability Density Function**).

First, we use baseline values in Table 2. For the deterministic model (1), we calculate the basic reproduction number

$$R_{0}=\frac{\bar{b}m\lambda \mu \delta }{d\left(a\mu +\epsilon \gamma \right)\left(r\delta +q\eta \right)}=6.7093>1,$$

and the infection steady state *Q*∗ = (1.0701, 1.2162, 1.0094, 4.0925, 0.6740). From Corollary 3.2, we obtain that the infection steady state is globally asymptotically stable if *R*_0_ > 1.

For the stochastic model (17), we calculate that

$$R_{0}^{s}=\frac{\bar{b}m\lambda \mu \delta e^{\frac{\sigma^{2}}{16\rho }}}{d\left(a\mu +\epsilon \gamma \right)\left(r\delta +q\eta \right)}=6.7608>1.$$

**Fig. 5:**
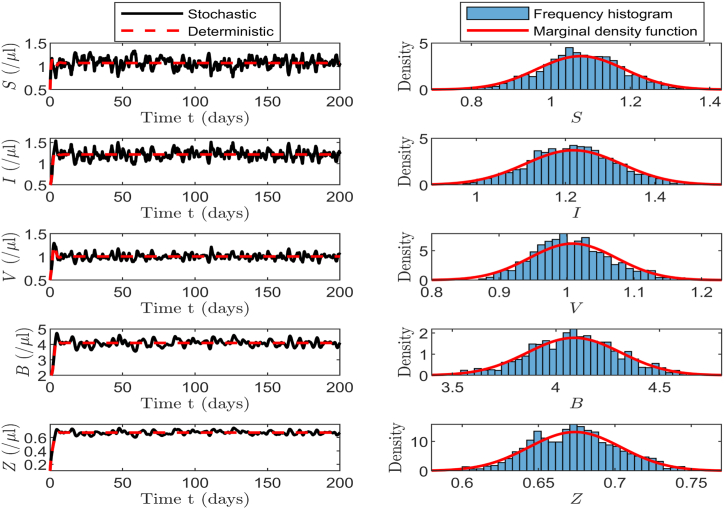
The left column displays the solutions of *S*, *I*, *V*, *B* and *Z* in both the stochastic model (17) and deterministic model (1). On the right column, the corresponding frequency histograms and probability density curves of the stochastic model are presented.

Based on the left panel of Fig. 5, we observe that the infection steady state in deterministic model (1) is globally asymptotically stable, while the corresponding solutions of stochastic model (17) remain at a certain level and fluctuate within a controllable range, which corresponds to the existence of the stationary distribution as stated in Theorem 4.1. The right panel of Fig. 5 shows the frequency histogram and probability density function curves for each population in the stochastic model, revealing that the fitted curves closely align with the characteristics of the normal distribution.

According to baseline values in Table 2, we calculate that *b*_4_ ≠ 0, *b*_9_ ≠ 0 and *R*_0_ > 1 hold, and the conditions of Theorem 5.1 are satisfied. Thereafter, the solution *X*(*t*) of system (17) approximately has a normal probability density function $\Phi \left(X\right)∼N_{6}\left({\tilde{Q}}^{*},\Sigma_{0}\right)$, where the quasi-steady state ${\tilde{Q}}^{*}=\left(1.0701,1.2162,1.0094,4.0925,0.6740,0\right)$, and

$$\Sigma_{0}=\left(\begin{array}{llllll} 0.0087 & -0.0083 & -0.0042 & -0.0105 & -0.0017 & -0.0110\\ -0.0083 & 0.0081 & 0.0043 & 0.0086 & 0.0014 & 0.0109\\ -0.0042 & 0.0043 & 0.0029 & 0.0033 & 0.0006 & 0.0034\\ -0.0105 & 0.0086 & 0.0033 & 0.0356 & 0.0047 & 0.0029\\ -0.0017 & 0.0014 & 0.0006 & 0.0047 & 0.0006 & 0.0008\\ -0.0110 & 0.0109 & 0.0034 & 0.0029 & 0.0008 & 0.0612 \end{array}\right).$$

Then, the marginal probability density functions for each population are denoted as

$$\begin{array}{l} \Phi_{1}\left(S\right)=4.2862e^{-57.7151{\left(S-1.0701\right)}^{2}},\\ \Phi_{2}\left(I\right)=4.4288e^{-61.6207{\left(I-1.2162\right)}^{2}},\\ \Phi_{3}\left(V\right)=7.3999e^{-172.0305{\left(V-1.0094\right)}^{2}},\\ \Phi_{4}\left(B\right)=2.1137e^{-14.0361{\left(B-4.0925\right)}^{2}},\\ \Phi_{5}\left(Z\right)=15.7467e^{-778.9823{\left(Z-0.6740\right)}^{2}},\\ \Phi_{6}\left(x\right)=1.6120e^{-8.1633x^{2}}. \end{array}$$

**Fig. 6:**
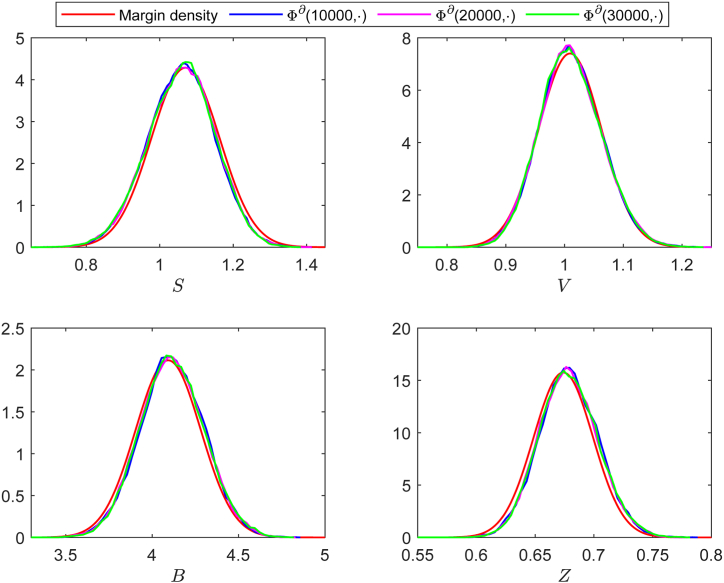
Simulation results of marginal density curve and the corresponding frequency histogram fitting curves at 10000, 20000, and 30000 iterations, respectively. Here, Φ^*∂*^(10000, ⋅), Φ^*∂*^(20000, ⋅), and Φ^*∂*^(30000, ⋅) represent the histogram fitting curves of each population at the corresponding iteration points, respectively.

We plot the marginal density function curves for each population, along with the frequency histogram fitting curves corresponding to the 10000, 20000, and 30000 iterations. As shown in Fig. 6, the marginal density curves for each population closely match the histogram fitting curves from the other three iterations, indicating that our method exhibits excellent fitting performance. Fig. 6 further illustrates that different numbers of iterations yield the same fitting results, indicating that the probability density function is not only local but also global.

Example 2 (**Influence of Volatility Intensity** ***σ***).

In this part, we select baseline values in Table 2 for the simulation to investigate the effects of different noise intensities (*σ* = 0.5, *σ* = 1, and *σ* = 1.5) on the number of CHIKV particles, B-cells and CTLs. We then calculate the corresponding values of $R_{0}^{s}$, which are 6.7355, 6.8149, and 6.9493, respectively. According to Theorem 4.1, these three scenarios guarantee the existence of the stationary distribution for system (17).

**Fig. 7:**
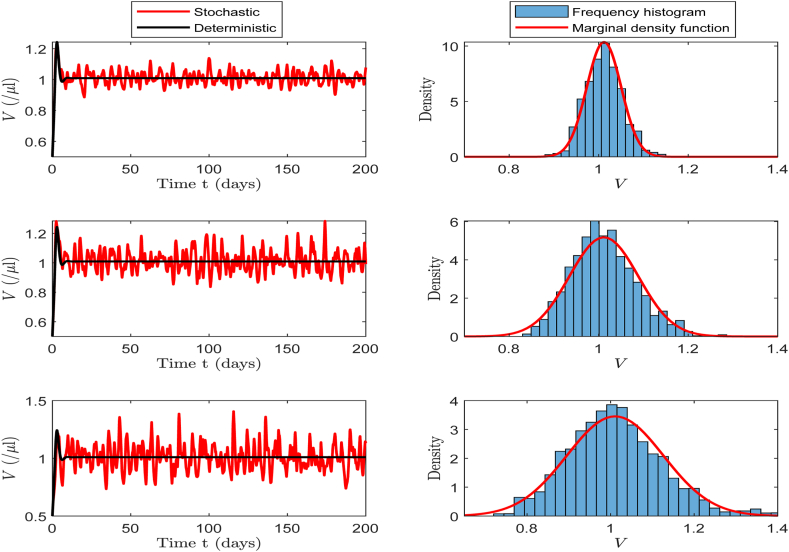
The left side of the figure shows the oscillations of CHIKV particles at different *σ* values of 0.5, 1.0 and 1.5, and the right side presents the corresponding frequency histogram and probability density curve.

**Fig. 8:**
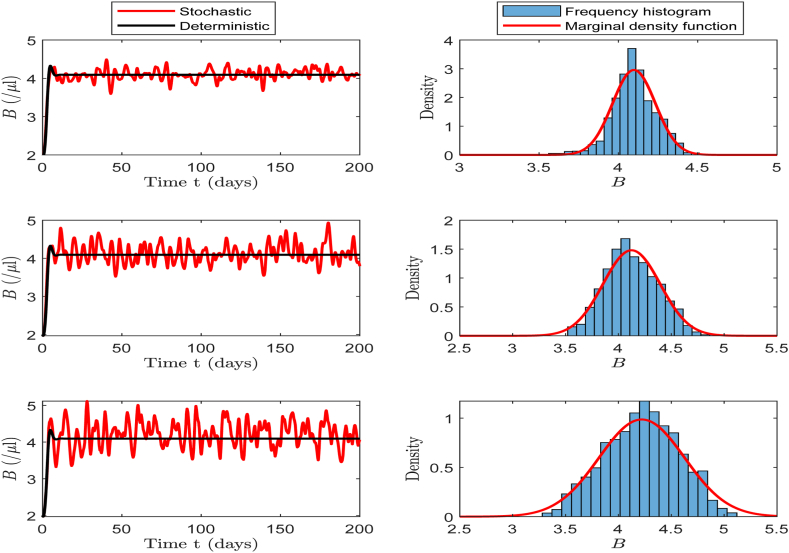
The left side of the figure shows the oscillations of B-cells at different *σ* values of 0.5, 1.0 and 1.5, and the right side presents the corresponding frequency histogram and probability density curve.

**Fig. 9:**
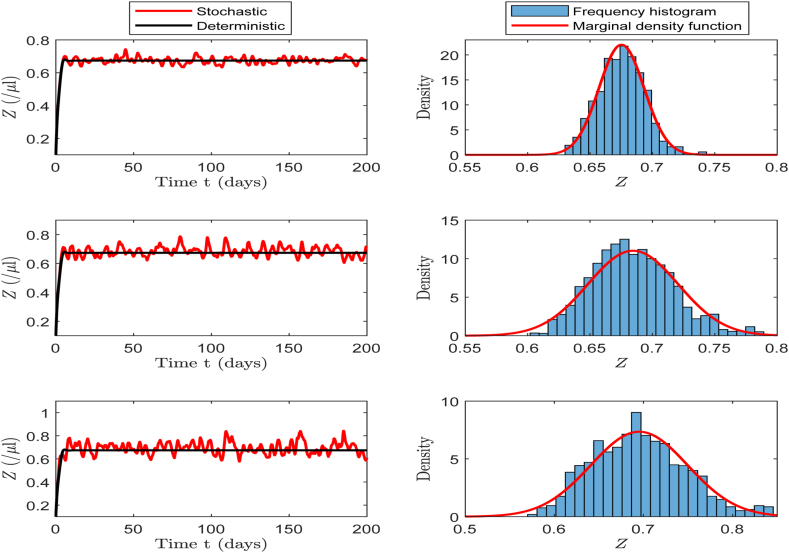
The left side of the figure shows the oscillations of CTLs at different *σ* values of 0.5, 1.0 and 1.5, and the right side presents the corresponding frequency histogram and probability density curve.

In the left panels of Fig. 7, Fig. 8, Fig. 9, we can clearly observe that as the noise intensity *σ* increases, the amplitudes of CHIKV particles, B-cells, and CTLs all exhibit an increasing trend. The panels on the right show the frequency histograms and probability density curves corresponding to each population. These figures indicate that when the noise intensity is low, the solution of the system exhibits only minor fluctuation near the infection steady state, presenting a relatively stable state. However, as the noise intensity increases, the oscillation amplitude of the system's solution increases significantly, further illustrating the notable impact of noise on the system's dynamic behavior.

Example 3 **(Extinction)**.

By selecting $\bar{b}=0.1$, *m* = 1.5, *δ* = 1.12, *μ* = 0.8, *ρ* = 1, and *σ* = 0.1, while keeping the other parameter values consistent with baseline values in Table 2, we obtain

$$R_{0}^{e}=\sqrt{R_{0}}+\frac{\sqrt{R_{0}}\left(r\delta +q\eta \right){\left(e^{\frac{\sigma^{2}}{\rho }}-2e^{\frac{\sigma^{2}}{4\rho }}+1\right)}^{\frac{1}{2}}}{\delta \min\left\{a+\frac{\epsilon \gamma }{\mu },r+\frac{q\eta }{\delta }\right\}}=0.8451<1.$$

**Fig. 10:**
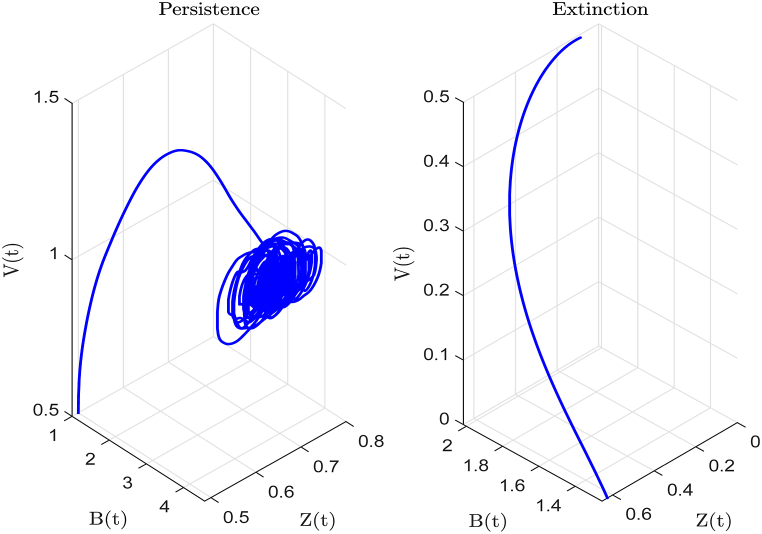
The left side curve tends towards a non-zero state, while the right side curve tends towards zero.

According to Theorem 4.2, when $R_{0}^{e}<1$, the CHIKV virus undergoes an exponential decline leading to eventual extinction. The right hand side of Fig. 10 demonstrates that the virus will ultimately be eradicated. Conversely, the left hand side of Fig. 10 illustrates the persistence of *V*, *B*, and *Z* using the values provided in baseline values of Table 2.

Example 4 (**The Effect of Key Parameters on Critical Conditions**).

As established in the uncertainty and sensitivity analysis of Section 6, the noise intensity *σ*, the reversion rate *ρ*, and the average incidence rate $\bar{b}$ critically influence the magnitude of the stochastic reproduction number $R_{0}^{s}$ and the dynamic behavior of the model. Therefore, we select appropriate parameter ranges to visually demonstrate the impact of *σ*, *ρ*, and $\bar{b}$ on the model's dynamical behavior.

**Fig. 11:**
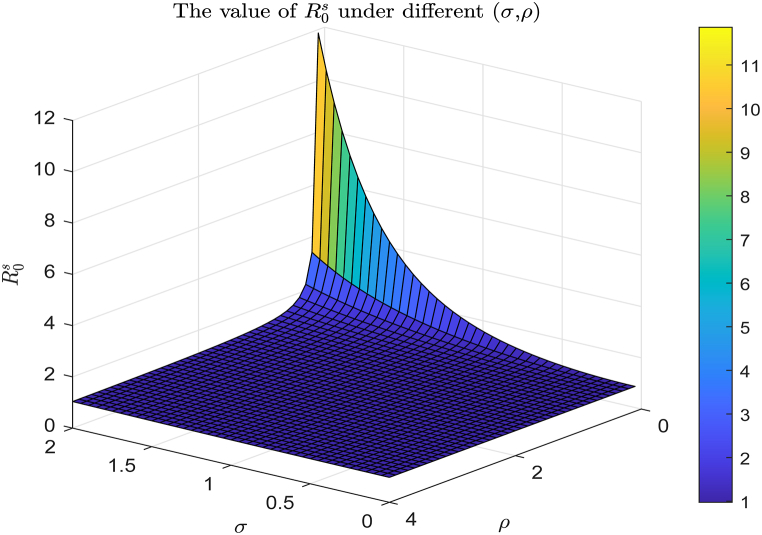
The trend of $R_{0}^{s}$ value for (*σ*, *ρ*) varying within the ranges [0, 2] × [0, 4], with the remaining data being selected from baseline values in Table 2.

**Fig. 12:**
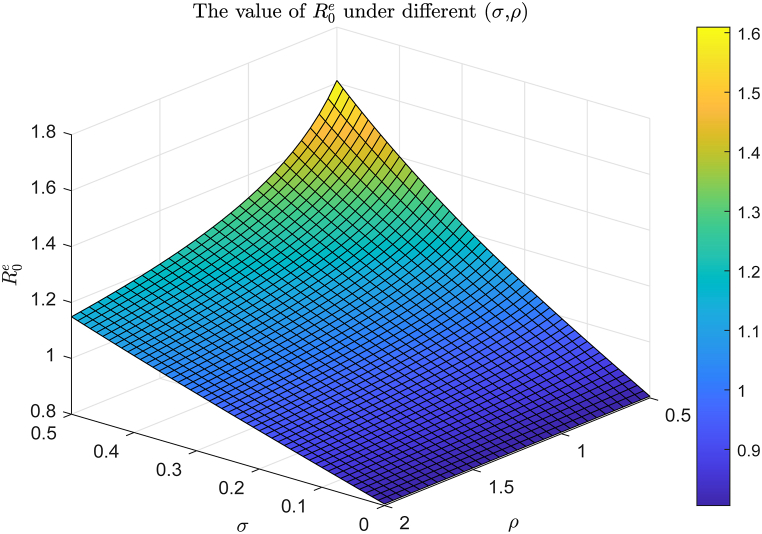
The trend of $R_{0}^{e}$ values for (*σ*, *ρ*) varying within the ranges [0, 0.5] × [0.5, 2], with the remaining data being selected from baseline values in Table 2.

From Fig. 11, Fig. 12, we observe that as the value of *ρ* increases and the value of *σ* decreases, both $R_{0}^{s}$ and $R_{0}^{e}$ exhibit decreasing trends. This implies that a decrease in noise intensity and an increase in the inversion rate both lead to a significant increase in the probability of viral extinction. This phenomenon is not only of great theoretical significance but also aligns with the actual infection characteristics of virus within the host.

**Fig. 13:**
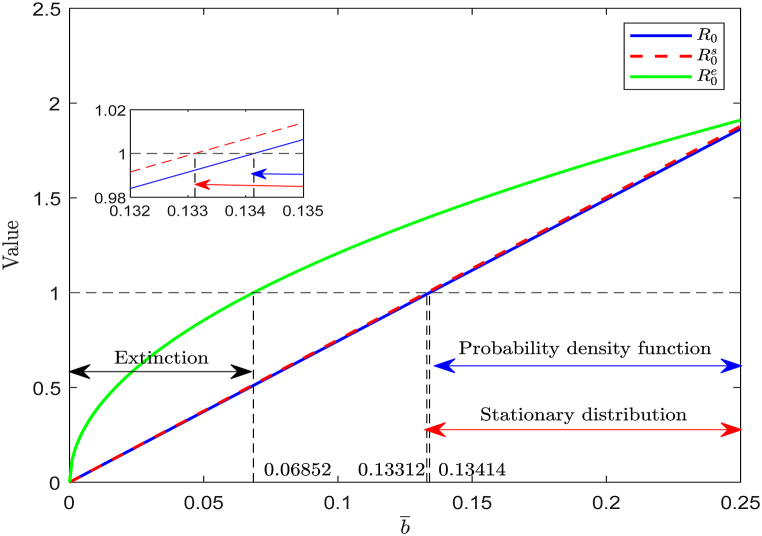
Evolution of *R*_0_, $R_{0}^{s}$, $R_{0}^{e}$ with respect to $\bar{b}\in \left(0,0.25\right)$.

Fig. 13 demonstrates the trends of *R*_0_, $R_{0}^{s}$ and $R_{0}^{e}$ with respect to varying values of $\bar{b}$. Combining Theorem 4.1, Theorem 4.2,and 5.1, we observe that.

(i) when $\bar{b}\in \left(0,0.06852\right)$, $R_{0}^{e}<1$, indicating that the virus will eventually become extinct.
(ii) when $\bar{b}\in \left(0.13312,0.13414\right)$, *R*_0_ < 1 and $R_{0}^{s}>1$, implying that system (17) possesses the stationary distribution.
(iii) when $\bar{b}\in \left(0.13414,0.25\right)$, $R_{0}^{s}>R_{0}>1$, system (17) not only exhibits the stationary distribution but also follows the normal distribution.
(iv) when $\bar{b}\in \left(0.06852,0.13312\right)$, the behavior of system (17) remains uncertain, and it may lead to either viral persistence or extinction.

Example 5 (**Influence of Incidence Rate** $\bar{b}$).

The magnitude of the incidence rate impacts the virus infection, subsequently influencing the development trend of infection in the model. Consequently, we will investigate how varying the average incidence rate affect target cells, CHIKV particles, B-cells and CTLs. The selected incidence rates $\bar{b}$ are 0.1, 0.5, 1 and 1.5, which correspond to the basic reproduction numbers *R*_0_ of 0.7455, 3.7274, 7.4547, and 11.1821, respectively. Notably, the corresponding $R_{0}^{s}$ values are 0.7512, 3.7560, 7.5120, and 11.2681, respectively.

**Fig. 14:**
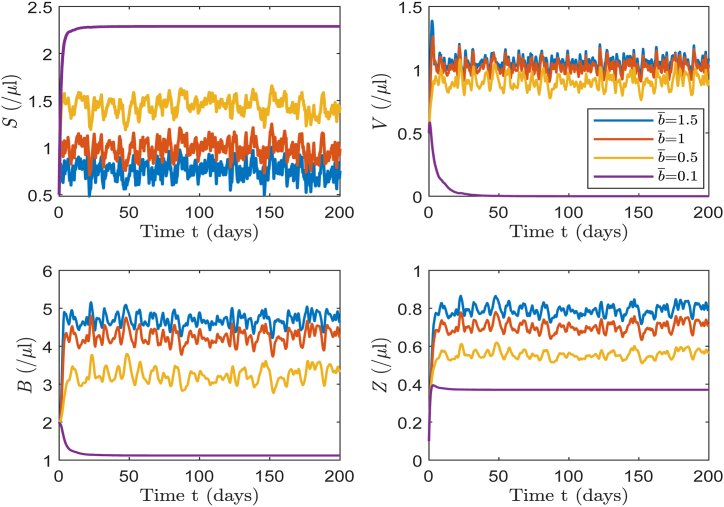
The solutions of *S*, *V*, *B* and *Z* for different average incidence rates of 0.1, 0.5, 1 and 1.5. Other parameter values are sourced from baseline values in Table 2.

The simulation in Fig. 14 reveals that reducing the average incidence rate results in the decay of CHIKV particles, B-cells, and CTLs, and the recovery of target cells. At lower average incidence rates, CHIKV levels decline rapidly toward zero. These results strongly suggest that lowering the average incidence rate is an effective strategy for CHIKV control, and it aligns consistently with actual infection dynamics.

Example 6 (**Mean First Passage Time**).

A core issue in studying the infection process is the transition time of host cells from the initial state (*S*(0), *I*(0), *V*(0), *B*(0), *Z*(0)) to the persistent infected state (*S*(*θ*_1_), *I*(*θ*_1_), *V*(*θ*_1_), *B*(*θ*_1_), *Z*(*θ*_1_)). This transition time can be quantified by the First Passage Time (FPT) and its statistical mean, the Mean First Passage Time (MFPT), which is one of the key indicators for quantifying such switching behavior in stochastic systems (Duan, 2015; Yang, Wang, et al., 2022).

By selecting the same parameters as in Example 1, we obtain (*S*(*θ*_1_), *I*(*θ*_1_), *V*(*θ*_1_), *B*(*θ*_1_), *Z*(*θ*_1_)) =(1.0701, 1.2162, 1.0094, 4.0925, 0.6740). Define the FPT from the initial state to the persistent state as $\tilde{s}$, where

$$\tilde{s}=\inf\left\{t:\;I\left(t\right)>1.2162,\;V\left(t\right)>1.0094\right\}.$$

Taking the average of the FPT, we generate MFPT = $E\left(\tilde{s}\right)$. Applying the Monte Carlo method, if $I\left(\tilde{v}\Delta t\right)>I\left(\theta_{1}\right)$ and $V\left(\tilde{v}\Delta t\right)>V\left(\theta_{1}\right)$, then $\tilde{s}=\tilde{v}\Delta t$ and

$$\text{MFPT}=\frac{\sum_{i=1}^{N}{\tilde{v}}_{i}\Delta t}{N}.$$

Under the fixed parameter *N* = 2000, Fig. 15 displays the trend of MFPT versus noise intensity *σ* ∈ [0.1, 2] under different reversion rates *ρ* = 1, 2, and 3. The MFPT exhibits a decreasing trend with both decreasing the reversion rate and increasing the noise intensity.

**Fig. 15:**
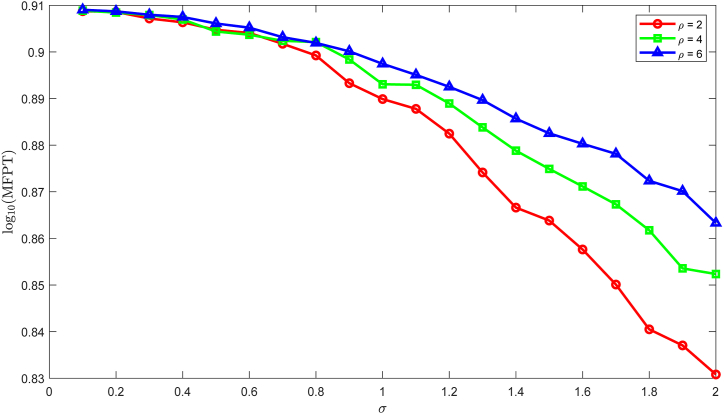
The MFPT fo transferring from the initial state (*S*(0), *I*(0), *V*(0), *B*(0), *Z*(0))=(2.2885, 10^−4^, 10^−4^, 1.2518, 0.6250) to the persistent state.

## 8 Conclusion

In this study, we propose a kind of CHIKV infection model incorporating dual immune responses in both deterministic and stochastic frameworks, where the stochastic perturbation is introduced into the general incidence rate by the log-normal OU process. We establish the existence of the unique global positive solution and analyze the system's behavior near the infection-free and infected steady states. Notably, the Lyapunov function constructed for the stochastic model generalizes to the corresponding deterministic case. This unified approach enables us to derive global asymptotic stability for the deterministic system at its steady states, thereby revealing fundamental connections between the stochastic and deterministic dynamics.

Theoretically, by constructing appropriate Lyapunov functions and compact set, we demonstrate that the system has at least one stationary distribution when $R_{0}^{s}>1$. Using spectral radius analysis, we conclude that the virus exhibits exponential extinction when $R_{0}^{e}<1$. Furthermore, we derive an approximate expression for the probability density function around the quasi-steady state of the stochastic model, thereby providing a theoretical basis for understanding the CHIKV dynamics.

Sensitivity analysis using LHS and PRCC methods reveals two principal findings: (i) The constant input rate *λ* and incidence rate $\bar{b}$ exhibit the most substantial influence on target cell, B-cell, and CTL populations dynamics. (ii) Parameters *λ* and $\bar{b}$ consistently exhibit positive correlations with the stochastic reproduction number $R_{0}^{s}$, while *d* demonstrates a negative effect. The impact of other parameters is noise-dependent: under low noise intensity, both *ρ* and *σ* show negligible influence, with key parameters aligning with the deterministic scenario; under high noise intensity, however, *ρ* and *σ* become key factors, significantly increasing the probability of $R_{0}^{s}>1$.

Numerically, we obtain the following key findings: (i) The marginal probability density function aligns closely with the corresponding frequency histogram, with higher noise intensity amplifying fluctuations for each population. (ii) Decreasing the noise intensity (*σ*) or increasing the reversion rate (*ρ*) significantly enhances the probability of viral extinction. (iii) A reduction in the average incidence rate ($\bar{b}$) leads to decreased numbers of CHIKV particles, B cells, and CTLs, accompanied by an increase in target cells. Notably, under low infection rates, the viral load declines rapidly toward zero. (iv) The MFPT from the initial state to the persistently infected state decreases with both decreasing the reversion rate and increasing the noise intensity.

Research on viral dynamical models that include latent infection and time delay is also of great significance. For the deterministic model, some researchers have investigated CHIKV models that include latent infection, active infection, and two types of distributed delay (Alade, 2021b; Elaiw et al., 2018). Wang and Liu (Wang & Liu, 2017) further proposed a CHIKV model that incorporated time delay and humoral immune response, exploring the impact of immune delay on the dynamical behavior of the model. However, stochastic CHIKV models involving the OU process, latent infection and time delays are relatively scarce, and will be a direction for our future research.

## CRediT authorship contribution statement

**Jingze Ma:** Writing – review & editing, Writing – original draft, Validation, Software, Formal analysis. **Yan Wang:** Writing – review & editing, Supervision, Methodology, Investigation, Conceptualization.

## Data availability

The data that support the findings of this study are available within the article.

## Conflict of interest

The authors have no conflicts to disclose.

## Appendix A Proof of Theorem 2.1

For any given initial value *X*_0_, the coefficients of system (5) satisfy the local Lipschitz continuity, ensuring that the system has a unique positive solution *X*(*t*) on interval $\left[0,\tau_{0}\right]$, where *τ*_0_ is the explosion time. If *τ*_0_ = *∞*, then a positive solution exists on [0, *∞*], indicating that *X*(*t*) is a global positive solution. Next, we will demonstrate this hypothesis.

We define a sufficiently large positive constant *n*_0_ such that *X*(*t*) lies within the interval [1/*n*_0_, *n*_0_]. For each integer *n* ≥ *n*_0_, the stopping time is defined as

$$\tau_{n}=\inf\left\{0<t<\tau_{0}\left|\;\min\left\{X_{i}\left(t\right)\right\}\leq \frac{1}{n}\right)\text{ or }\max\left\{X_{i}\left(t\right)\right\}\geq n,\;i=1,2,\ldots ,5\right\}.$$

From the definition of *τ*_*n*_, we can see that *τ*_*n*_ increases with *n* and that *τ*_*∞*_ ≤ *τ*_0_. If *τ*_*∞*_ = *∞*, then it follows that *τ*_0_ = *∞*. Conversely, if *τ*_*∞*_ < *∞*, there exists a positive constant $\tilde{T}$ and a value *ϖ* ∈ (0, 1) such that $P\left\{\tau_{\infty }\leq \tilde{T}\right\}>\varpi$. Consequently, there exists an integer *n*_1_ ≥ *n*_0_ such that for all *n* ≥ *n*_1_, it holds that $P\left\{\tau_{n}\leq \tilde{T}\right\}\geq \varpi$.

Define a non-negative *C*^2^-function *C*: $R_{+}^{5}\times R\to R_{+}$ as follows:

$$\begin{array}{l} C\left(X\right)=S-1-\ln\;S+I-1-\ln\;I+V-1-\ln\;V+\frac{q}{c}(B-1-\ln\;B)+\frac{\epsilon }{\omega }\left(Z-1-\ln\;Z\right)+e^{x}-1-x. \end{array}$$

Applying It$\hat{o}$’s formula (Mao, 2008) and introducing the differential operator L, we have

(A.1) $$\begin{array}{l} LC=\lambda -dS-\frac{\lambda }{S}+d+\bar{b}e^{x}F\left(V\right)-aI-\frac{\bar{b}e^{x}SF\left(V\right)}{I}+a+\epsilon Z+mI-rV-m\frac{I}{V}+r+qB+\frac{q\eta }{c}\\ \;\;\;\;\;\;\;\;\;\;-\frac{q\delta B}{c}-\frac{q\eta }{cB}-qV+\frac{q\delta }{c}+\frac{\epsilon \gamma }{\omega }-\frac{\epsilon \mu }{\omega }Z-\frac{\epsilon \gamma }{\omega Z}-\epsilon I+\frac{\epsilon \mu }{\omega }-\rho x\left(e^{x}-1\right)+\frac{\sigma^{2}}{2}e^{x}\\ \;\;\;\;\;\;\leq \lambda +d+\bar{b}e^{x}F\left(V\right)+a+\epsilon Z+mI+r+qB+\frac{q\eta }{c}+\frac{q\delta }{c}+\frac{\epsilon \gamma }{\omega }+\frac{\epsilon \mu }{\omega }-\rho x\left(e^{x}-1\right)+\frac{\sigma^{2}}{2}e^{x}. \end{array}$$

In addition, in view of system (5), one gets

$$S\left(t\right)\leq \left\{\begin{array}{ll} S\left(0\right),\; & \text{if }S\left(0\right)>\frac{\lambda }{d}\\ \frac{\lambda }{d},\; & \text{if }S\left(0\right)\leq \frac{\lambda }{d} \end{array}\right)\leq N_{1}≔\max\left\{S\left(0\right),\frac{\lambda }{d}\right\},$$

$$\begin{array}{l} S\left(t\right)+I(t)\leq \left\{\begin{array}{ll} S\left(0\right)+I\left(0\right),\; & \text{if }S\left(0\right)+I\left(0\right)>M_{1}\\ M_{1},\; & \text{if }S\left(0\right)+I\left(0\right)\leq M_{1} \end{array}\right)\leq N_{2}≔\max\left\{S\left(0\right)+I\left(0\right),M_{1}\right\}, \end{array}$$

$$V\left(t\right)\leq \left\{\begin{array}{ll} V\left(0\right),\; & \text{if }V\left(0\right)>M_{2}\\ M_{2},\; & \text{if }V\left(0\right)\leq M_{2} \end{array}\right)\leq N_{3}≔\max\left\{V\left(0\right),M_{2}\right\}.$$

$$B\left(t\right)\leq \left\{\begin{array}{ll} B\left(0\right),\; & \text{if }B\left(0\right)>M_{3}\\ M_{3},\; & \text{if }B\left(0\right)\leq M_{3} \end{array}\right)\leq N_{4}≔\max\left\{B\left(0\right),M_{3}\right\},$$

$$Z\left(t\right)\leq \left\{\begin{array}{ll} Z\left(0\right),\; & \text{if }Z\left(0\right)>M_{4}\\ M_{4},\; & \text{if }Z\left(0\right)\leq M_{4} \end{array}\right)\leq N_{5}≔\max\left\{Z\left(0\right),M_{4}\right\},$$

where the expression for *M*_*i*_, *i* = 1, 2, 3, 4 is given by (6) and (7), respectively.

From condition $\left(A_{3}\right)$ it follows that

$$\frac{F\left(V\right)}{V}\leq \lim_{V\to 0}\frac{F\left(V\right)}{V}=\lim_{V\to 0}\frac{F\left(V\right)-F\left(0\right)}{V}=F^{\prime }\left(0\right),$$

that is

(A.2) $$F\left(V\right)\leq F^{\prime }\left(0\right)V.$$

Substituting inequality (A.2) into (A.1), we obtain

$$LC\leq \lambda +d+a+r+mN_{2}+qN_{4}+\epsilon N_{5}+\frac{q}{c}\left(\eta +\delta \right)+\frac{\epsilon }{\omega }\left(\gamma +\mu \right)+f\left(x\right),$$

where

$$f\left(x\right)=\left(\bar{b}F^{\prime }\left(0\right)N_{3}+\frac{\sigma^{2}}{2}\right)e^{x}-\rho x\left(e^{x}-1\right).$$

By analyzing the function *f*(*x*), we derive that *f*(−*∞*) = −*∞*, *f*(+*∞*) = −*∞*. Thereafter

$$LC\leq \lambda +d+a+r+mN_{2}+qN_{4}+\epsilon N_{5}+\frac{q}{c}\left(\eta +\delta \right)+\frac{\epsilon }{\omega }\left(\gamma +\mu \right)+\sup_{x\in R}\left\{f\left(x\right)\right\}≔K,$$

where *K* is a positive constant. The remaining proof is similar to the proof process in literature (Dong et al., 2025). This completes the proof.

## Appendix B Preliminaries

Lemma Appendix B.1 *(See Theorem 2.2 in* (Du et al., 2016)*) Suppose that there is a bounded closed domain* $D\subset R^{d}$*, for any initial value* $X\left(0\right)\in R^{d}$*, if*

$${\mathrm{lim inf}}_{t\to \infty }\frac{1}{t}\int_{0}^{t}P\left(\tau ,X\left(0\right),D\right)d\tau >0\text{ a.s. }$$

*wh**ere* $P\left(\tau ,X\left(0\right),\cdot \right)$ *denotes the transition probability of* *X*(*τ*)*. Then system* (5) *possesses a solution that has the Feller property and at least one stationary distribution* *π*(⋅) *on* $R^{d}$.
Lemma Appendix B.2 *For the mean-reverting OU process* (4)*, we have*

(i)

(B.1) $$\begin{array}{l} \lim_{t\to +\infty }\frac{1}{t}\int_{0}^{t}e^{nx\left(\tau \right)}d\tau =\int_{-\infty }^{+\infty }e^{nx}\pi \left(x\right)dx=\frac{\sqrt{\rho }}{\sigma \sqrt{\pi }}\int_{-\infty }^{+\infty }e^{nx-\frac{\rho }{\sigma^{2}}x^{2}}dx\\ \;\;\;\;\;\;\;\;\;\;\;\;\;\;\;\;\;\;\;\;\;\;\;\;\;\;\;\;\;\;\;\;\;\;\;\;\;\;\;=\frac{\sqrt{\rho }}{\sigma \sqrt{\pi }}\int_{-\infty }^{+\infty }e^{-{\left(\frac{\sqrt{\rho }}{\sigma }x-\frac{n\sigma }{2\sqrt{\rho }}\right)}^{2}+\frac{n^{2}\sigma^{2}}{4\rho }}dx=\frac{1}{\sqrt{\pi }}\int_{-\infty }^{+\infty }e^{-t^{2}+\frac{n^{2}\sigma^{2}}{4\rho }}dt=e^{\frac{n^{2}\sigma^{2}}{4\rho }}, \end{array}$$

*w**here* $\pi \left(x\right)=\frac{\sqrt{\rho }}{\sigma \sqrt{\pi }}e^{-\frac{\rho }{\sigma^{2}}x^{2}}$ *and* *n* *is a positive constant.*

(ii) *Using the H*$\ddot{o}$*lder inequality,*

(B.2) $$\begin{array}{ll} \lim_{t\to \infty }\frac{1}{t}\int_{0}^{t}\left|e^{x\left(\tau \right)}-1\right|d\tau & \leq \lim_{t\to \infty }\frac{1}{t}{\left(\int_{0}^{t}1^{2}d\tau \right)}^{\frac{1}{2}}{\left(\int_{0}^{t}|e^{x\left(\tau \right)}-1{|}^{2}d\tau \right)}^{\frac{1}{2}}\\ & =\lim_{t\to \infty }{\left(\frac{1}{t}\int_{0}^{t}{\left(e^{x\left(\tau \right)}-1\right)}^{2}d\tau \right)}^{\frac{1}{2}}\\ & =\bar{b}{\left(e^{\frac{\sigma^{2}}{\rho }}-2e^{\frac{\sigma^{2}}{4\rho }}+1\right)}^{\frac{1}{2}}. \end{array}$$

Lemma Appendix B.3 (Ma et al., 2015)*) The characteristic polynomial of matrix* *A* *is defined as* *φ*_*A*_(*κ*) = *κ*^*n*^ + *a*_1_*κ*^*n*−1^ + ⋯ + *a*_*n*−1_*κ* + *a*_*n*_*, then* *A* *is called a Hurwitz matrix if and only if the real part of all eigenvalues of* *A* *are negative, i.e.,* $\left|H_{A}^{\left(k\right)}\right|>0,k=1,2,\ldots ,n,$ *where*

$$H_{A}^{\left(k\right)}=\left|\begin{array}{ccccc} a_{1} & a_{3} & a_{5} & \cdots & a_{2k-1}\\ 1 & a_{2} & a_{4} & \cdots & a_{2k-2}\\ 0 & a_{1} & a_{3} & \cdots & a_{2k-3}\\ 0 & 1 & a_{2} & \cdots & a_{2k-4}\\ \cdots & \cdots & \cdots & \cdots & \cdots \\ 0 & 0 & 0 & \cdots & a_{k} \end{array}\right|,$$

*a*_*k*_ = 0*, for* *k* > *n**. Furthermore, the necessary conditions for* *A* *to be a Hurwitz matrix are as follows (i)* *a*_*k*_ > 0, *k* = 1, 2, …, *n**, (ii)* *a*_*k*_*a*_*k*+1_ > *a*_*k*−1_*a*_*k*+2_*,* *k* = 1, 2, …, *n* − 2*, and* *a*_0_ = 1*. In this case, we write* $A\in \bar{RH}\left(n\right)$. *Moreover, as referenced in* (Han & Jiang, 2023)*, we use the shorthand notation* $H_{A}^{\left(6\right)}\left(i,\eta_{0}\right)$ *to represent a matrix by replacing only the* *i**-th column of* $H_{A}^{\left(6\right)}$ *with* ***η***_0_*, where*. $\eta_{0}={\left(0,0,0,0,0,1\right)}^{\text{T}}.$
Lemma Appendix B.4 *(*(Han & Jiang, 2023)*) For a six-dimensional real algebraic equation*

$$I_{0}^{2}+A\Sigma +\Sigma A^{\text{T}}=0,$$

*where* *I*_0_ = diag(1, 0, 0, 0, 0, 0), Σ *is a symmetric matrix, and*

$$A=\left(\begin{array}{llllll} -d_{1} & -d_{2} & -d_{3} & -d_{4} & -d_{5} & -d_{6}\\ 1 & 0 & 0 & 0 & 0 & 0\\ 0 & 1 & 0 & 0 & 0 & 0\\ 0 & 0 & 1 & 0 & 0 & 0\\ 0 & 0 & 0 & 1 & 0 & 0\\ 0 & 0 & 0 & 0 & 1 & 0 \end{array}\right).$$

*If* $\left|H_{A}^{\left(k\right)}\right|>0$ *for* *k* = 1, 2, …, 6*, then* $A\in \bar{RH}\left(6\right)$*, thus*

$$\Sigma =\left(\begin{array}{llllll} h_{11} & 0 & -h_{22} & 0 & h_{33} & 0\\ 0 & h_{22} & 0 & -h_{33} & 0 & h_{44}\\ -h_{22} & 0 & h_{33} & 0 & -h_{44} & 0\\ 0 & -h_{33} & 0 & h_{44} & 0 & -h_{55}\\ h_{33} & 0 & -h_{44} & 0 & h_{55} & 0\\ 0 & h_{44} & 0 & -h_{55} & 0 & h_{66} \end{array}\right),$$

*where* $h_{ii}=\frac{{\left(-1\right)}^{i+1}\left|H_{A}\left(i,\eta_{1}\right)\right|}{2\left|H_{A}\right|},\;i=1,2,\ldots ,6$*, where* ***η***_1_ = (1, 0, 0, 0, 0, 0)*. Note that* Σ *is a positive definite matrix.*

## Appendix C Proof of Theorem 4.1

The proof is divided into three steps: (i) construct a non-negative *C*^2^-function; (ii) define a compact set; and (iii) demonstrate the existence of a stationary distribution.

**Step 1. Constructing a non-negative** ***C*^2^****-function**

By condition ($A_{3}$) as well as the Lagrange Median Theorem, it follows that

$$\frac{\frac{V}{F\left(V\right)}-\frac{1}{F^{\prime }\left(0\right)}}{V-0}={\left({\left(\frac{V}{F\left(V\right)}\right)}^{\prime }\right|}_{V=\xi_{3}}\leq K_{0},\;\xi_{3}\in \left(0,V\right).$$

Then, we have

$$\frac{V}{F\left(V\right)}-\frac{1}{F^{\prime }\left(0\right)}\leq K_{0}V.$$

From (B.1) in Lemma Appendix B.2, we have

(C.1) $$\lim_{t\to +\infty }\frac{1}{t}\int_{0}^{t}e^{2x\left(\tau \right)}d\tau =e^{\frac{\sigma^{2}}{\rho }},\lim_{t\to +\infty }\frac{1}{t}\int_{0}^{t}e^{\frac{1}{4}x\left(\tau \right)}d\tau =e^{\frac{\sigma^{2}}{64\rho }}.$$

Using Young's inequality, we obtain

$$e^{x}\leq \theta e^{2x}+\frac{1}{4\theta },$$

where *θ* > 0.

Define a *C*^2^-function as

$$V_{1}=-\ln\;V-c_{1}\ln\;I-c_{2}\ln\;S+\frac{c_{1}\epsilon }{\mu }Z+\frac{q}{\delta }B+\left[\frac{c}{\delta }+\frac{c_{2}F^{\prime }\left(0\right)\bar{b}}{4\theta r}+\frac{c_{3}K_{0}}{r}\right]V.$$

where *c*_1_, *c*_2_ and *c*_3_ are positive constants, whose values are provided later. Applying It$\hat{o}$’s formula, and combining it with the above inequalities, we obtain

$$\begin{array}{ll} LV_{1}= & -m\frac{I}{V}+r+qB+c_{1}\left[-\frac{\bar{b}e^{x}SF\left(V\right)}{I}+a+\epsilon Z\right]+c_{2}\left[d+\bar{b}e^{x}F\left(V\right)-\frac{\lambda }{S}\right]+\frac{c_{1}\epsilon }{\mu }\left(\gamma +\omega IZ-\mu Z\right)\\ & +\frac{q}{\delta }\left(\eta +cBV-\delta B\right)+\frac{c}{\delta }\left(mI-rV-qVB\right)-c_{3}\frac{V}{F\left(V\right)}+c_{3}\left[\frac{V}{F\left(\left(V\right)\right)}-\frac{1}{F^{\prime }\left(0\right)}\right]+c_{3}\frac{1}{F^{\prime }\left(0\right)}+\left[\frac{c_{2}F^{\prime }\left(0\right)\bar{b}}{4\theta r}+\frac{c_{3}K_{0}}{r}\right]\left(mI-rV-qVB\right)\\ & \leq -\left[m\frac{I}{V}+c_{1}\frac{\bar{b}e^{x}SF\left(V\right)}{I}+c_{2}\frac{\lambda }{S}+c_{3}\frac{V}{F\left(V\right)}\right]+c_{1}\left(a+\frac{\epsilon \gamma }{\mu }\right)+c_{2}d+c_{3}\frac{1}{F^{\prime }\left(0\right)}+r+\frac{q\eta }{\delta }\\ & +c_{2}\bar{b}e^{x}F^{\prime }\left(0\right)V+\frac{c_{1}\epsilon \omega }{\mu }IZ+c_{3}\left[\frac{V}{F\left(V\right)}-\frac{1}{F^{\prime }\left(0\right)}\right]+\frac{cm}{\delta }I+\left[\frac{c_{2}F^{\prime }\left(0\right)\bar{b}}{4\theta r}+\frac{c_{3}K_{0}}{r}\right]\left(mI-rV\right)\\ & \leq -4\sqrt[{4}]{\bar{b}m\lambda c_{1}c_{2}c_{3}e^{x}}+c_{1}\left(a+\frac{\epsilon \gamma }{\mu }\right)+c_{2}d+c_{3}\frac{1}{F^{\prime }\left(0\right)}+r+\frac{q\eta }{\delta }+c_{2}F^{\prime }\left(0\right)\bar{b}\left(\theta e^{2x}+\frac{1}{4\theta }\right)V+\frac{c_{1}\epsilon \omega }{\mu }M_{4}I\\ & +c_{3}K_{0}V+\frac{cm}{\delta }I+\left[\frac{c_{2}F^{\prime }\left(0\right)\bar{b}}{4\theta r}+\frac{c_{3}K_{0}}{r}\right]\left(mI-rV\right)\\ & \leq -4\sqrt[{4}]{\bar{b}m\lambda c_{1}c_{2}c_{3}e^{x}}+c_{1}\left(a+\frac{\epsilon \gamma }{\mu }\right)+c_{2}d+c_{3}\frac{1}{F^{\prime }\left(0\right)}+r+\frac{q\eta }{\delta }+\left(\frac{c_{1}\epsilon \omega M_{4}}{\mu }+\frac{cm}{\delta }\right)I\\ & +c_{2}F^{\prime }\left(0\right)\bar{b}\theta e^{2x}M_{2}+\left[\frac{c_{2}F^{\prime }\left(0\right)\bar{b}}{4\theta r}+\frac{c_{3}K_{0}}{r}\right]mI\\ & =-4\sqrt[{4}]{\bar{b}m\lambda c_{1}c_{2}c_{3}e^{\frac{\sigma^{2}}{16\rho }}}+c_{1}\left(a+\frac{\epsilon \gamma }{\mu }\right)+c_{2}\left[d+F^{\prime }\left(0\right)\bar{b}\theta M_{2}e^{\frac{\sigma^{2}}{\rho }}\right]+c_{3}\frac{1}{F^{\prime }\left(0\right)}+r+\frac{q\eta }{\delta }+K_{1}I+h_{1}\left(x\right)+h_{2}\left(x\right), \end{array}$$

where

$$\begin{array}{l} K_{1}=\frac{c_{1}\epsilon \omega M_{4}}{\mu }+\frac{c_{2}F^{'}\left(0\right)\bar{b}m}{4\theta r}+\frac{c_{3}K_{0}m}{r}+\frac{cm}{\delta },\;h_{1}\left(x\right)=c_{2}F^{'}\left(0\right)\bar{b}\theta M_{2}\left(e^{2x}-e^{\frac{\sigma^{2}}{\rho }}\right),\\ h_{2}\left(x\right)=4\left(\sqrt[{4}]{m\lambda \bar{b}c_{1}c_{2}c_{3}e^{\frac{\sigma^{2}}{16\rho }}}-\sqrt[{4}]{m\lambda \bar{b}c_{1}c_{2}c_{3}e^{x}}\right). \end{array}$$

Let

$$\begin{array}{ll} & c_{1}=\frac{\bar{b}m\lambda \mu^{2}e^{\frac{\sigma^{2}}{16\rho }}F^{\prime }\left(0\right)}{\left[d+F^{\prime }\left(0\right)\bar{b}\theta M_{2}e^{\frac{\sigma^{2}}{\rho }}\right]{\left(a\mu +\epsilon \gamma \right)}^{2}},\;c_{2}=\frac{\bar{b}m\lambda \mu e^{\frac{\sigma^{2}}{16\rho }}F^{\prime }\left(0\right)}{{\left[d+F^{\prime }\left(0\right)\bar{b}\theta M_{2}e^{\frac{\sigma^{2}}{\rho }}\right]}^{2}\left(a\mu +\epsilon \gamma \right)},\\ & c_{3}=\frac{\bar{b}m\lambda \mu e^{\frac{\sigma^{2}}{16\rho }}{\left[F^{\prime }\left(0\right)\right]}^{2}}{\left[d+F^{\prime }\left(0\right)\bar{b}\theta M_{2}e^{\frac{\sigma^{2}}{\rho }}\right]\left(a\mu +\epsilon \gamma \right)}. \end{array}$$

Then, we obtain

(C.2) $$\begin{array}{ll} LV_{1}\leq -\frac{\bar{b}m\lambda \mu e^{\frac{\sigma^{2}}{16\rho }}F^{'}\left(0\right)}{\left(d+F^{'}\left(0\right)\bar{b}\theta M_{2}e^{\frac{\sigma^{2}}{\rho }}\right)\left(a\mu +\epsilon \gamma \right)}+r+\frac{q\eta }{\delta }+K_{1}I+h_{1}\left(x\right)+h_{2}\left(x\right)=-\Lambda_{1}+K_{1}I+h_{1}\left(x\right)+h_{2}\left(x\right), & \; \end{array}$$

where

$$\Lambda_{1}=\frac{r\delta +q\eta }{\delta }\left(R_{0}^{s}\left(\theta \right)-1\right),$$

and

$$R_{0}^{s}\left(\theta \right)=\frac{\bar{b}m\lambda \mu \delta e^{\frac{\sigma^{2}}{16\rho }}F^{\prime }\left(0\right)}{\left(d+F^{\prime }\left(0\right)\bar{b}\theta M_{2}e^{\frac{\sigma^{2}}{\rho }}\right)\left(a\mu +\epsilon \gamma \right)\left(r\delta +q\eta \right)}.$$

We further have

$$R_{0}^{s}=\lim_{\theta \to 0}R_{0}^{s}\left(\theta \right)=\frac{\bar{b}m\lambda \mu \delta e^{\frac{\sigma^{2}}{16\rho }}F^{\prime }\left(0\right)}{d\left(a\mu +\epsilon \gamma \right)\left(r\delta +q\eta \right)}.$$

Next, define

$$V_{2}=-\ln\;S,\;V_{3}=-\ln\;V,\;V_{4}=-\ln\;B,\;V_{5}=-\ln\;Z,\;V_{6}=e^{x}-1-x.$$

Then applying It$\hat{o}$’s formula, we have

(C.3) $$\begin{array}{l} LV_{2}=d+\bar{b}e^{x}F\left(V\right)-\frac{\lambda }{S}\leq -\frac{\lambda }{S}+d+\bar{b}e^{x}F^{'}\left(0\right)M_{2},\\ LV_{3}=-m\frac{I}{V}+r+qB\leq -m\frac{I}{V}+r+qM_{3},\\ LV_{4}=-\frac{\eta }{B}-cV+\delta \leq -\frac{\eta }{B}+\delta ,\\ LV_{5}=-\frac{\gamma }{Z}-\omega I+\mu \leq -\frac{\gamma }{Z}+\mu ,\\ LV_{6}=-\rho x\left(e^{x}-1\right)+\frac{\sigma^{2}}{2}e^{x}. \end{array}$$

Finally, we define

(C.4) $$W\left(X\right)=MV_{1}+V_{2}+V_{3}+V_{4}+V_{5}+V_{6},$$

and *M* > 0 is a sufficiently large constant to be satisfied:

(C.5) $$-M\Lambda_{1}+K_{2}\leq -2,$$

where $K_{2}=\sup_{\begin{array}{c} x\in R \end{array}}\left\{\Lambda_{2}+h_{3}\left(x\right)\right\}<\infty$, Λ_2_ = *d* + *r* + *qM*_3_ + *δ* + *μ*, $h_{3}\left(x\right)=\left[\bar{b}F^{\prime }\left(0\right)M_{2}+\frac{\sigma^{2}}{2}\right]e^{x}-\rho x\left(e^{x}-1\right)$. Combining (C.2), (C.3) and (C.4), we obtain

$$\begin{array}{l} LW\leq -M\Lambda_{1}+MK_{1}I+Mh_{1}\left(x\right)+Mh_{2}\left(x\right)-\frac{\lambda }{S}-m\frac{I}{V}-\frac{\eta }{B}-\frac{\gamma }{Z}+\Lambda_{2}+h_{3}\left(x\right)\\ \;\;\;\;\;\;\;=G\left(X\right)+Mh_{1}\left(x\right)+Mh_{2}\left(x\right), \end{array}$$

where $G\left(X\right)=-M\Lambda_{1}+MK_{1}I-\frac{\lambda }{S}-m\frac{I}{V}-\frac{\eta }{B}-\frac{\gamma }{Z}+\Lambda_{2}+h_{3}\left(x\right)$.

**Step 2. Constructing a compact set** Now we construct a compact set *D* of the following form

$$D=\left\{X\in \Gamma |S\geq \upsilon ,I\geq \upsilon ,V\geq \upsilon ,B\geq \upsilon ,Z\geq \upsilon ,\upsilon \leq e^{x}\leq \frac{1}{\upsilon }\right\},$$

where *υ* ∈ (0, 1) is a sufficiently small constant such that the following inequalities hold,

(C.6) $$MK_{1}\upsilon \leq 1,$$

(C.7) $$-\min\left\{\frac{\lambda }{\upsilon },\frac{m}{\upsilon },\frac{\eta }{\upsilon },\frac{\gamma }{\upsilon }\right]+MK_{1}M_{1}\leq 1,$$

(C.8) $$\frac{\rho \ln\;\upsilon }{2\upsilon }+K_{3}\leq -1,$$

(C.9) $$\frac{\rho \ln\;\upsilon }{2}+K_{4}\leq -1.$$

Next, we partition the set Γ∖*D* into seven subsets $D_{i}^{c}\left(i=1,\ldots ,7\right)$, and $D^{c}={⋃}_{i=1}^{7}D_{i}^{c}$, where

$$\begin{array}{ll} & D_{1}^{c}=\left\{X\in \Gamma |I<\upsilon \right\},\;D_{2}^{c}=\left\{X\in \Gamma |S<\upsilon \right\},\;D_{3}^{c}=\left\{X\in \Gamma |V<\upsilon^{2},I⩾\upsilon \right\},\;D_{4}^{c}=\left\{X\in \Gamma |B<\upsilon \right\},\\ & D_{5}^{c}=\left\{X\in \Gamma |Z<\upsilon \right\},\;D_{6}^{c}=\left\{X\in \Gamma |x>\ln\frac{1}{\upsilon }\right\},\;D_{7}^{c}=\left\{X\in \Gamma |x<\ln\;\upsilon \right\}. \end{array}$$

Case 1 If $X\in D_{1}^{c}$, it follows from (C.5) and (C.6) that

$$G\left(X\right)\leq -M\Lambda_{1}+MK_{1}I+\Lambda_{2}+h_{3}\left(x\right)\leq MK_{1}\upsilon -2\leq -1.$$

Case 2 If $X\in D_{2}^{c}$, according to (C.5) and (C.7), we get

$$G\left(X\right)\leq -\frac{\lambda }{S}+MK_{1}I-2\leq -\frac{\lambda }{\upsilon }+MK_{1}M_{1}-2\leq -1.$$

Case 3 If $X\in D_{3}^{c}$, from (C.5) and (C.7) we have

$$G\left(X\right)\leq -m\frac{I}{V}+MK_{1}I-2\leq -\frac{m}{\upsilon }+MK_{1}M_{1}-2\leq -1.$$

Case 4 If $X\in D_{4}^{c}$, based on (C.5) and (C.7) we obtain

$$G\left(X\right)\leq -\frac{\eta }{B}+MK_{1}I-2\leq -\frac{\eta }{\upsilon }+MK_{1}M_{1}-2\leq -1.$$

Case 5 If $X\in D_{5}^{c}$, according to (C.5) and (C.7), we get

$$G\left(X\right)\leq -\frac{\gamma }{Z}+MK_{1}I-2\leq -\frac{\gamma }{\upsilon }+MK_{1}M_{1}-2\leq -1.$$

Case 6 If $X\in D_{6}^{c}$, in view of (C.8), we obtain

$$\begin{array}{l} G\left(X\right)\leq -\frac{\rho }{2}xe^{x}+MK_{1}I+\Lambda_{2}+\left(\bar{b}F^{'}\left(0\right)M_{2}+\frac{\sigma^{2}}{2}\right)e^{x}-\rho x\left(\frac{1}{2}e^{x}-1\right)\\ \;\;\;\;\;\;\;\;\;\leq -\frac{\rho \ln\frac{1}{\upsilon }}{2\upsilon }+MK_{1}M_{1}+\Lambda_{2}+\left(\bar{b}F^{'}\left(0\right)M_{2}+\frac{\sigma^{2}}{2}\right)e^{x}-\rho x\left(\frac{1}{2}e^{x}-1\right)\\ \;\;\;\;\;\;\;\;\;\leq \frac{\rho \ln\;\upsilon }{2\upsilon }+K_{3}\leq -1, \end{array}$$

where

$$K_{3}≔\sup_{\begin{array}{c} x\in R \end{array}}\left\{MK_{1}M_{1}+\Lambda_{2}+\left(\bar{b}F^{\prime }\left(0\right)M_{2}+\frac{\sigma^{2}}{2}\right)e^{x}-\rho x\left(\frac{1}{2}e^{x}-1\right)\right\}.$$

Case 7 If $X\in D_{7}^{c}$, it follows from (C.9) that

$$\begin{array}{l} G\left(X\right)\leq \frac{\rho }{2}x+MK_{1}I+\Lambda_{2}+\left(\bar{b}F^{'}\left(0\right)M_{2}+\frac{\sigma^{2}}{2}\right)e^{x}-\rho x\left(e^{x}-\frac{1}{2}\right)\\ \;\;\;\;\;\;\;\;\;\leq \frac{\rho \ln\;\upsilon }{2}+MK_{1}M_{1}+\Lambda_{2}+\left(\bar{b}F^{'}\left(0\right)M_{2}+\frac{\sigma^{2}}{2}\right)e^{x}-\rho x\left(e^{x}-\frac{1}{2}\right)\\ \;\;\;\;\;\;\;\;\;\leq \frac{\rho \ln\;\upsilon }{2}+K_{4}\leq -1, \end{array}$$

where

$$K_{4}≔\sup_{\begin{array}{c} x\in R \end{array}}\left\{MK_{1}M_{1}+\Lambda_{2}+\left(\bar{b}F^{\prime }\left(0\right)M_{2}+\frac{\sigma^{2}}{2}\right)e^{x}-\rho x\left(e^{x}-\frac{1}{2}\right)\right\}.$$

In conclusion, we have

(C.10) $$G\left(X\right)\leq -1\text{ for any }X\in D^{c}.$$

**Step 3. Existence of the stationary distribution**

Because *W* is a continuous function on Γ, there exists a point $\tilde{X_{0}}\in \Gamma$ such that $W\left(\tilde{X_{0}}\right)$ attains its minimum value. Then, we define a non-negative *C*^2^-function $\tilde{W}$ as follows:

$$\tilde{W}\left(X\right)=W\left(X\right)-W\left(\tilde{X_{0}}\right),$$

thus we obtain

$$L\tilde{W}\leq G\left(X\right)+Mh_{1}\left(x\right)+Mh_{2}\left(x\right).$$

Integrating both sides from 0 to *t* and then taking the expectation, we have

(C.11) $$0\leq \frac{E\tilde{W}\left[X\left(t\right)\right]}{t}\;=\frac{E\tilde{W}\left[X\left(0\right)\right]}{t}+\frac{1}{t}\int_{0}^{t}E\left[L\tilde{W}\left(X\left(\tau \right)\right)\right]d\tau \leq \frac{E\tilde{W}\left[X\left(0\right)\right]}{t}+\frac{1}{t}\int_{0}^{t}E\left[G\left(X\left(\tau \right)\right)\right]d\tau +4M\sqrt[{4}]{m\lambda \bar{b}c_{1}c_{2}c_{3}}E\left[e^{\frac{\sigma^{2}}{64\rho }}-\frac{1}{t}\int_{0}^{t}e^{\frac{x\left(\tau \right)}{4}}d\tau \right]+Mc_{2}F^{'}\left(0\right)\bar{b}\theta M_{2}E\left[\frac{1}{t}\int_{0}^{t}e^{2}x\left(\tau \right)d\tau -e^{\frac{\sigma^{2}}{\rho }}\right].$$

Based on the strong law of large numbers (Liptser, 1980) and Equation (C.1), we have

$$\lim_{t\to \infty }E\left[\frac{1}{t}\int_{0}^{t}e^{2x\left(\tau \right)}d\tau -e^{\frac{\sigma^{2}}{\rho }}\right]=0,\;\lim_{t\to \infty }E\left[e^{\frac{\sigma^{2}}{64\rho }}-\frac{1}{t}\int_{0}^{t}e^{\frac{x\left(\tau \right)}{4}}d\tau \right]=0,\;\;\text{ a.s. }$$

Taking the limit on both sides of (C.11), we obtain

(C.12) $$0\leq \lim_{t\to \infty }\frac{E\tilde{W}\left(X_{0}\right)}{t}+\lim_{t\to \infty }\frac{1}{t}\int_{0}^{t}E\left[G\left(X\left(\tau \right)\right)\right]d\tau =\lim_{t\to \infty }\frac{1}{t}\int_{0}^{t}E\left[G\left(X\left(\tau \right)\right)\right]d\tau ,\;\;\text{ a.s. }$$

In addition,

(C.13) $$G\left(X\right)\leq \tilde{K}<\infty ,\text{ for any }X\in \Gamma ,$$

where

$$\tilde{K}=\sup_{\begin{array}{c} X\in \Gamma \end{array}}\left\{-M\Lambda_{1}+MK_{1}I-\frac{\lambda }{S}-m\frac{I}{V}-\frac{\eta }{B}-\frac{\gamma }{Z}+\Lambda_{2}+h_{3}\left(x\right)\right\}<\infty .$$

However, from (C.10) and (C.13), we obtain

$$\lim_{t\to +\infty }\frac{1}{t}\int_{0}^{t}E\left[G\left(X\left(\tau \right)\right)\right]d\tau =\lim_{t\to +\infty }\frac{1}{t}\int_{0}^{t}E\left[G\left(X\left(\tau \right)\right)\right]1_{\left\{\left(X\left(\tau \right)\right)\in D\right\}}d\tau +\lim_{t\to +\infty }\frac{1}{t}\int_{0}^{t}E\left[G\left(X\left(\tau \right)\right)\right]1_{\left\{\left(X\left(\tau \right)\right)\in D^{c}\right\}}d\tau \leq \tilde{K}\lim_{t\to +\infty }\frac{1}{t}\int_{0}^{t}{\mathbb{P}}_{\left\{\left(X\left(\tau \right)\right)\in D\right\}}d\tau -\lim_{t\to +\infty }\frac{1}{t}\int_{0}^{t}{\mathbb{P}}_{\left\{\left(X\left(\tau \right)\right)\in D^{c}\right\}}d\tau \leq \left(\tilde{K}+1\right)\lim_{t\to +\infty }\frac{1}{t}\int_{0}^{t}{\mathbb{P}}_{\left\{\left(X\left(\tau \right)\right)\in D\right\}}d\tau -1.$$

Combining equation (C.12), we get

$$\lim_{t\to +\infty }\frac{1}{t}\int_{0}^{t}P_{\left\{\left(X\left(\tau \right)\right)\in D\right\}}d\tau \geq \frac{1}{\tilde{K}+1}>0,\;\text{a.s.}$$

By the property of the infimum, we obtain

$${\mathrm{lim inf}}_{t\to \infty }\frac{1}{t}\int_{0}^{t}P_{\left\{\left(X\left(\tau \right)\right)\in D\right\}}d\tau \geq \frac{1}{\tilde{K}+1}>0,\;\text{a.s.}$$

Using Fatou's lemma (Du et al., 2016), and then we get

$${\mathrm{lim inf}}_{t\to \infty }\frac{1}{t}\int_{0}^{t}P\left(\tau ,\left(X\left(\tau \right)\right),D\right)d\tau \geq \frac{1}{\tilde{K}+1}>0,\;\text{a.s.}$$

Consequently, considering Lemma Appendix B.1, we obtain that system (5) admits at least one stationary distribution *π*(⋅) with the Feller property on Γ. The proof is confirmed.

## Appendix D Proof of Theorem 4.2

To illustrate the extinction of the virus, we employ the spectral radius method. First, we define a matrix

$$\begin{array}{l} L=\left(\begin{array}{ll} 0 & \frac{\mu \bar{b}\lambda F^{\prime }\left(0\right)}{d\left(a\mu +\epsilon \gamma \right)}\\ \frac{m\delta }{r\delta +q\eta } & 0 \end{array}\right). \end{array}$$

For the nonsingular matrix *L*, there exists an eigenvector $\left(w_{1},w_{2}\right)$ of its eigenvalues such that

$$\left(w_{1},w_{2}\right)L=\sqrt{R_{0}}\left(w_{1},w_{2}\right),$$

where $\left(w_{1},w_{2}\right)=\left(1,\sqrt{R_{0}}\left(r\delta +q\eta \right)/m\delta \right)$.

Define a *C*^2^-function $U:R_{+}^{2}\to R_{+}$ as follows

$$U\left(I,V\right)=\phi_{1}I+\phi_{2}V,$$

where $\phi_{1}=\frac{\mu }{a\mu +\epsilon \gamma }w_{1}$, $\phi_{2}=\frac{\delta }{r\delta +q\eta }w_{2}$. Applying It$\hat{o}$’s formula to ln *U*, we have

(D.1) $$\begin{array}{ll} L\left(\ln\;U\right) & =\frac{1}{U}\left[\phi_{1}\left(bSF\left(V\right)-aI-\epsilon IZ\right)+\phi_{2}\left(mI-rV-qVB\right)\right]\\ & \leq \frac{1}{U}\left[\phi_{1}\bar{b}S_{0}F^{\prime }\left(0\right)V-\phi_{1}aI-\phi_{1}\epsilon IZ_{0}+\phi_{2}mI-\phi_{2}rV-\phi_{2}qVB_{0}\right]+\frac{1}{U}\left[\phi_{1}\left(b-\bar{b}\right)S_{0}F^{\prime }\left(0\right)V\right]\\ & \leq \frac{1}{U}\left[\phi_{1}\bar{b}\frac{\lambda }{d}F^{\prime }\left(0\right)V+\phi_{2}mI-\phi_{1}\frac{a\mu +\epsilon \gamma }{\mu }I-\phi_{2}\frac{r\delta +q\eta }{\delta }V\right]+\frac{\phi_{1}}{\phi_{2}}|b-\bar{b}|\frac{\lambda F^{\prime }\left(0\right)}{d}\\ & =\frac{1}{U}\left[\frac{\mu \bar{b}\lambda F^{\prime }\left(0\right)}{d\left(a\mu +\epsilon \gamma \right)}w_{1}V+\frac{m\delta }{r\delta +q\eta }w_{2}I-w_{1}I-w_{2}V\right]+\frac{\phi_{1}}{\phi_{2}}|b-\bar{b}|\frac{\lambda F^{\prime }\left(0\right)}{d}\\ & =\frac{1}{U}\left[\left(\frac{m\delta w_{2}}{r\delta +q\eta },\frac{\mu \bar{b}\lambda F^{\prime }\left(0\right)w_{1}}{d\left(a\mu +\epsilon \gamma \right)}\right)\left(\begin{array}{l} I\\ V \end{array}\right)-\left(w_{1},w_{2}\right)\left(\begin{array}{l} I\\ V \end{array}\right)\right]+\frac{\phi_{1}}{\phi_{2}}|b-\bar{b}|\frac{\lambda F^{\prime }\left(0\right)}{d}\\ & =\frac{1}{U}\left(\sqrt{R_{0}}-1\right)\left(w_{1},w_{2}\right)\left(\begin{array}{l} I\\ V \end{array}\right)+\frac{\phi_{1}}{\phi_{2}}|b-\bar{b}|\frac{\lambda F^{\prime }\left(0\right)}{d}\\ & =\left(\sqrt{R_{0}}-1\right)\frac{1}{\phi_{1}I+\phi_{2}V}\left[\left(a+\frac{\epsilon \gamma }{\mu }\right)\phi_{1}I+r+\frac{q\eta }{\delta }\phi_{2}V\right]+\frac{\sqrt{R_{0}}\left(r\delta +q\eta \right)}{\bar{b}\delta }|b-\bar{b}|\\ & \leq -\min\left\{a+\frac{\epsilon \gamma }{\mu },r+\frac{q\eta }{\delta }\right\}\left(1-\sqrt{R_{0}}\right)+\frac{\sqrt{R_{0}}\left(r\delta +q\eta \right)}{\bar{b}\delta }|b-\bar{b}|. \end{array}$$

Integrating (D.1) from 0 to *t*, then dividing by *t* on both sides, we obtain

$$\frac{\ln\;U\left(t\right)}{t}\leq \frac{\ln\;U\left(0\right)}{t}-\min\left\{a+\frac{\epsilon \gamma }{\mu },r+\frac{q\eta }{\delta }\right\}\left(1-\sqrt{R_{0}}\right)+\frac{\sqrt{R_{0}}\left(r\delta +q\eta \right)}{\bar{b}\delta t}\int_{0}^{t}|b\left(\tau \right)-\bar{b}|d\tau .$$

Taking the superior limit on both sides and referring to (B.2), we obtain:

$$\begin{array}{ll} {\mathrm{lim sup}}_{t\to \infty }\frac{\ln\;U\left(t\right)}{t} & \leq -\min\left\{a+\frac{\epsilon \gamma }{\mu },r+\frac{q\eta }{\delta }\right\}\left(1-\sqrt{R_{0}}\right)+\frac{\sqrt{R_{0}}\left(r\delta +q\eta \right)}{\bar{b}\delta }{\mathrm{lim sup}}_{t\to \infty }\frac{1}{t}\int_{0}^{t}|b\left(\tau \right)-\bar{b}|d\tau \\ & \leq -\min\left\{a+\frac{\epsilon \gamma }{\mu },r+\frac{q\eta }{\delta }\right\}\left(1-\sqrt{R_{0}}\right)+\frac{\sqrt{R_{0}}\left(r\delta +q\eta \right)}{\delta }{\left(e^{\frac{\sigma^{2}}{\rho }}-2e^{\frac{\sigma^{2}}{4\rho }}+1\right)}^{\frac{1}{2}}\\ & \leq -\min\left\{a+\frac{\epsilon \gamma }{\mu },r+\frac{q\eta }{\delta }\right\}\left(1-R_{0}^{e}\right). \end{array}$$

Clearly, if $R_{0}^{e}<1$, it concludes that

$$\lim_{t\to \infty }I\left(t\right)=0\text{ and }\lim_{t\to \infty }V\left(t\right)=0,\;\text{ a.s.}$$

In other words, the infected cells and CHIKV particles will become exponentially extinct with probability one. This completes this proof.

## Appendix E Proof of Theorem 5.1

Based on the results in reference (Yang, Zhang, & Jiang, 2022; Zhou et al., 2022), we conclude that the following algebraic equation determines the covariance matrix Σ_0_

(E.1) $$H_{0}^{2}+A_{0}\Sigma_{0}+\Sigma_{0}A_{0}^{\text{T}}=0.$$

In addition, we obtain the characteristic polynomial of *A*_0_ as follows:

$$\varphi_{A_{0}}\left(\kappa \right)=\left(\kappa +\rho \right)\varphi_{Q^{*}}\left(\kappa \right),$$

where $\varphi_{Q^{*}}\left(\kappa \right)$ is the characteristic polynomial of the Jacobi matrix corresponding to the infection steady state *Q*∗. In Corollary 3.2, we proved that under conditions $\left(A_{2}\right)$ and $\left(A_{3}\right)$, and *R*_0_ > 1, *Q*∗ is globally asymptotically stable. Therefore, all eigenvalues of matrix *A*_0_ have negative real parts. According to Lemma Appendix B.3, we know that $A_{0}\in \bar{RH}\left(6\right)$. Subsequently, we derive the explicit form of the covariance matrix Σ_0_.

Let $A_{1}=\left(I_{7}I_{6}I_{5}I_{4}I_{3}I_{2}I_{1}\right)A_{0}{\left(I_{7}I_{6}I_{5}I_{4}I_{3}I_{2}I_{1}\right)}^{-1}$, where

$$I_{1}=\left(\begin{array}{llllll} 0 & 0 & 0 & 0 & 0 & 1\\ 0 & 1 & 0 & 0 & 0 & 0\\ 0 & 0 & 1 & 0 & 0 & 0\\ 0 & 0 & 0 & 1 & 0 & 0\\ 0 & 0 & 0 & 0 & 1 & 0\\ 1 & 0 & 0 & 0 & 0 & 0 \end{array}\right),\;I_{2}=\left(\begin{array}{llllll} 1 & 0 & 0 & 0 & 0 & 0\\ 0 & 1 & 0 & 0 & 0 & 0\\ 0 & 0 & 1 & 0 & 0 & 0\\ 0 & 0 & 0 & 1 & 0 & 0\\ 0 & 0 & 0 & 0 & 1 & 0\\ 0 & 1 & 0 & 0 & 0 & 1 \end{array}\right),$$

$$I_{3}=\left(\begin{array}{llllll} 1 & 0 & 0 & 0 & 0 & 0\\ 0 & 1 & 0 & 0 & 0 & 0\\ 0 & 0 & 1 & 0 & 0 & 0\\ 0 & 0 & 0 & 1 & 0 & 0\\ 0 & 0 & -\frac{a_{52}}{a_{32}} & 0 & 1 & 0\\ 0 & 0 & 0 & 0 & 0 & 1 \end{array}\right),\;I_{4}=\left(\begin{array}{llllll} 1 & 0 & 0 & 0 & 0 & 0\\ 0 & 1 & 0 & 0 & 0 & 0\\ 0 & 0 & 1 & 0 & 0 & 0\\ 0 & 0 & 0 & 1 & 0 & 0\\ 0 & 0 & 0 & 0 & 1 & 0\\ 0 & 0 & -\frac{b_{1}}{a_{32}} & 0 & 0 & 1 \end{array}\right),$$

$$I_{5}=\left(\begin{array}{llllll} 1 & 0 & 0 & 0 & 0 & 0\\ 0 & 1 & 0 & 0 & 0 & 0\\ 0 & 0 & 1 & 0 & 0 & 0\\ 0 & 0 & 0 & 1 & 0 & 0\\ 0 & 0 & 0 & -\frac{b_{2}}{a_{43}} & 1 & 0\\ 0 & 0 & 0 & 0 & 0 & 1 \end{array}\right),\;I_{6}=\left(\begin{array}{llllll} 1 & 0 & 0 & 0 & 0 & 0\\ 0 & 1 & 0 & 0 & 0 & 0\\ 0 & 0 & 1 & 0 & 0 & 0\\ 0 & 0 & 0 & 1 & 0 & 0\\ 0 & 0 & 0 & 0 & 1 & 0\\ 0 & 0 & 0 & -\frac{b_{3}}{a_{43}} & 0 & 1 \end{array}\right),$$

$$I_{7}=\left(\begin{array}{llllll} 1 & 0 & 0 & 0 & 0 & 0\\ 0 & 1 & 0 & 0 & 0 & 0\\ 0 & 0 & 1 & 0 & 0 & 0\\ 0 & 0 & 0 & 1 & 0 & 0\\ 0 & 0 & 0 & 0 & 1 & 0\\ 0 & 0 & 0 & 0 & -\frac{b_{5}}{b_{4}} & 1 \end{array}\right),$$

where

$$\begin{array}{ll} & b_{1}=d-a-\epsilon Z^{*},\;b_{2}=\frac{a_{52}\left(a_{33}-a_{55}\right)}{a_{32}},\;b_{3}=\frac{b_{1}\left(a_{21}-a_{11}+a_{33}\right)-a_{25}a_{52}}{a_{32}},\\ & b_{4}=\frac{a_{52}}{a_{32}a_{43}}\left[\left(a_{33}-a_{55}\right)\left(a_{44}-a_{55}\right)+a_{34}a_{43}\right],\;b_{5}=\frac{a_{34}b_{1}}{a_{32}}-\frac{a_{25}a_{52}}{a_{32}a_{43}}\left(a_{33}-a_{55}\right)+\frac{b_{3}}{a_{43}}\left(a_{44}+a_{21}-a_{11}\right),\\ & b_{6}=a_{23}-\frac{a_{25}a_{52}-a_{21}b_{1}}{a_{32}},\;b_{7}=-\frac{a_{25}a_{52}}{a_{32}a_{43}}\left(a_{33}-a_{55}\right)+\frac{a_{21}b_{3}}{a_{43}},\;b_{8}=\frac{b_{5}}{b_{4}}\left(a_{55}+a_{21}-a_{11}\right)-a_{25}, \end{array}$$

and assuming that *b*_4_ ≠ 0 and *b*_8_ ≠ 0.

By calculation, we obtain

$$\begin{array}{l} A_{1}=\left(\begin{array}{llllll} -\rho & 0 & 0 & 0 & 0 & 0\\ a_{16} & -a_{21}-a_{22} & b_{6} & b_{7} & \frac{b_{5}a_{21}}{b_{4}}-a_{25} & a_{21}\\ 0 & a_{32} & -a_{33} & -a_{34} & 0 & 0\\ 0 & 0 & a_{43} & -a_{44} & 0 & 0\\ 0 & 0 & 0 & b_{4} & -a_{55} & 0\\ 0 & 0 & 0 & 0 & b_{8} & a_{21}-a_{11} \end{array}\right). \end{array}$$

Then, we can equivalently transform equation (E.1) into

(E.2) $$H_{1}^{2}+A_{1}\Sigma_{1}+\Sigma_{1}A_{1}^{\text{T}}=0,$$

where

$$\begin{array}{ll} H_{1}^{2}= & \left(I_{7}I_{6}I_{5}I_{4}I_{3}I_{2}I_{1}\right)H_{0}^{2}{\left(I_{7}I_{6}I_{5}I_{4}I_{3}I_{2}I_{1}\right)}^{\text{T}}=diag\left(\sigma^{2},0,0,0,0,0\right)=\sigma^{2}I_{0},\\ \Sigma_{1}= & \left(I_{7}I_{6}I_{5}I_{4}I_{3}I_{2}I_{1}\right)\Sigma_{0}{\left(I_{7}I_{6}I_{5}I_{4}I_{3}I_{2}I_{1}\right)}^{\text{T}}. \end{array}$$

Let $A_{2}=T_{0}A_{1}T_{0}^{-1}$, where the standardized transformation matrix *T*_0_ is given by

$$T_{0}=\left(\begin{array}{l} \eta_{0}{\left(A_{1}\right)}^{5}\\ \eta_{0}{\left(A_{1}\right)}^{4}\\ \eta_{0}{\left(A_{1}\right)}^{3}\\ \eta_{0}{\left(A_{1}\right)}^{2}\\ \eta_{0}A_{1}\\ \eta_{0} \end{array}\right).$$

By simple calculation, we have

$$\begin{array}{l} A_{2}=\left(\begin{array}{llllll} -d_{1} & -d_{2} & -d_{3} & -d_{4} & -d_{5} & -d_{6}\\ 1 & 0 & 0 & 0 & 0 & 0\\ 0 & 1 & 0 & 0 & 0 & 0\\ 0 & 0 & 1 & 0 & 0 & 0\\ 0 & 0 & 0 & 1 & 0 & 0\\ 0 & 0 & 0 & 0 & 1 & 0 \end{array}\right). \end{array}$$

Actually, *d*_*i*_ (*i* = 1, …, 6) are the coefficients of the characteristic polynomial of *A*_1_, which results from

$$\varphi_{A_{1}}\left(\kappa \right)=\kappa^{6}+\overset{6}{\underset{i=1}{\sum }}d_{i}\kappa^{6-i}.$$

From the knowledge of advanced algebra, we know that similarity transformations do not change the eigenvalues of a matrix. Based on $A_{0}\in \bar{RH}\left(6\right)$, we obtain $A_{1}\in \bar{RH}\left(6\right)$, and subsequently derive $A_{2}\in \bar{RH}\left(6\right)$. Then equation (E.2) can be transformed into

$$I_{0}^{2}+A_{2}\left(\frac{1}{k^{2}}T_{0}\Sigma_{1}T_{0}^{\text{T}}\right)+\left(\frac{1}{k^{2}}T_{0}\Sigma_{1}T_{0}^{\text{T}}\right)A_{2}^{\text{T}}=0,$$

where *k* = *a*_16_*a*_32_*a*_43_*b*_4_*b*_8_*σ*.

By Lemma Appendix B.4 and equation (E.2), the covariance matrix is given by

(E.3) $$\begin{array}{l} \frac{1}{k^{2}}T_{0}\Sigma_{1}T_{0}^{\text{T}}=\left(\begin{array}{llllll} h_{11} & 0 & -h_{22} & 0 & h_{33} & 0\\ 0 & h_{22} & 0 & -h_{33} & 0 & h_{44}\\ -h_{22} & 0 & h_{33} & 0 & -h_{44} & 0\\ 0 & -h_{33} & 0 & h_{44} & 0 & -h_{55}\\ h_{33} & 0 & -h_{44} & 0 & h_{55} & 0\\ 0 & h_{44} & 0 & -h_{55} & 0 & h_{66} \end{array}\right)≔\Xi . \end{array}$$

where

$$h_{ii}=\frac{{\left(-1\right)}^{i+1}\left|H_{A_{2}}\left(i,\eta_{1}\right)\right|}{2\left|H_{A_{2}}\right|},i=1,\ldots ,6.$$

Note that Ξ is positive definite matrix, thus $\Sigma_{1}=k^{2}T_{0}^{-1}\Xi {\left(T_{0}^{-1}\right)}^{\text{T}}$ is also a positive definite matrix. Moreover, it is important to note that $\Sigma_{0}={\left(I_{7}I_{6}I_{5}I_{4}I_{3}I_{2}I_{1}\right)}^{-1}\Sigma_{1}{\left[{\left(I_{7}I_{6}I_{5}I_{4}I_{3}I_{2}I_{1}\right)}^{\text{T}}\right]}^{-1}$ is positive definite. Furthermore, the density function Φ(*X*) of system (5) around the quasi-steady state $\tilde{Q^{*}}$ satisfies

$$\Phi \left(X\right)={\left(2\pi \right)}^{-3}|\Sigma_{0}{|}^{-\frac{1}{2}}e^{-\frac{1}{2}\left(X-\tilde{Q^{*}}\right)\Sigma_{0}^{-1}{\left(X-\tilde{Q^{*}}\right)}^{\text{T}}}.$$
